# Radial Data Visualization-Based Step-by-Step Eliminative Algorithm to Predict Colorectal Cancer Patients’ Response to FOLFOX Therapy

**DOI:** 10.3390/ijms252212149

**Published:** 2024-11-12

**Authors:** Jakub Kryczka, Rafał Adam Bachorz, Jolanta Kryczka, Joanna Boncela

**Affiliations:** 1 Laboratory of Cell Signaling, Institute of Medical Biology, Polish Academy of Sciences, 106 Lodowa St., 93-232 Lodz, Poland; jboncela@cbm.pan.pl; 2 Laboratory of Molecular Modeling, Institute of Medical Biology, Polish Academy of Sciences, 106 Lodowa St., 93-232 Lodz, Poland; rbachorz@cbm.pan.pl; 3 Department of Biomedicine and Genetics, Medical University of Lodz, 92-213 Lodz, Poland; jolanta.kryczka@umed.lodz.pl

**Keywords:** CRC, chemoresistance, FOLFOX, oxaliplatin, predictive algorithm, radial data visualization

## Abstract

Application of the FOLFOX scheme to colorectal cancer (CRC) patients often results in the development of chemo-resistance, leading to therapy failure. This study aimed to develop a functional and easy-to-use algorithm to predict patients’ response to FOLFOX treatment. Transcriptomic data of CRC patient’s samples treated with FOLFOX were downloaded from the Gene Expression Omnibus database (GSE83129, GSE28702, GSE69657, GSE19860 and GSE41568). Comparing the expression of top up- and downregulated genes in FOLFOX responder and non-responder patients’ groups, we selected 30 potential markers that were used to create a step-by-step eliminative procedure based on modified radial data visualization, which depicts the interplay between the expression level of chosen attributes (genes) to locate data points in low-dimensional space. Our analysis proved that FOLFOX-resistant CRC samples are predominantly characterized by upregulated expression levels of *TMEM182* and *MCM9* and downregulated *LRRFIP1.* Additionally, the procedure developed based on expression levels of *TMEM182*, *MCM9*, *LRRFIP1*, *LAMP1*, *FAM161A*, *KLHL36*, *ETV5*, *RNF168*, *SRSF11*, *NCKAP5*, *CRTAP*, *VAMP2*, *ZBTB49* and *RIMBP2* proved to be capable in predicting FOLFOX therapy response. In conclusion, our approach can give a unique insight into clinical decision-making regarding therapy scheme administration, potentially increasing patients’ survival and, consequently, medical futility due to incorrect therapy application.

## 1. Introduction

Even though surgical excision of the early primary tumor with proper histopathologic margins is considered the best treatment for most solid tumors, including colorectal cancer (CRC), once the cancer metastasis takes place, surgery for removing the tumor will no longer be a fully effective treatment, as it has been reported that the rate of relapse after resection of primary CRC and colorectal metastases ranges from 20 to 68% [1,2]. In such cases, other types of anti-cancer therapies are needed, and chemotherapy (including neoadjuvant chemotherapy) remains one of the basic treatment modalities [3]. Since early 2000, one of the most prominent chemotherapy schemes used for CRC is FOLFOX, composed of folinic acid (FnA), 5-fluorouracil (5-Fu), and oxaliplatin (OxP) [4,5]. Unfortunately, CRC cells often develop resistance to chemotherapy components. This phenomenon is one of the most significant factors in overall therapy failure, leading to a low patient survival rate [6]. Interestingly, even though the mechanisms of chemotherapy have been extensively explored, we lack clinically useful markers of oxaliplatin or FOLFOX resistance [6,7,8,9].

Since the 1960s, with the introduction of computer-based pattern analysis, it has become clear that the implementation of artificial intelligence and its ability to recognize and evaluate large data via pre-trained algorithms (“a systematic procedure that produces, in a finite number of steps, the answer to a question or the solution of a problem”) may enhance oncological therapy decision-making [10,11]. However, an insufficient number of clinically valuable data and the low availability of hardware computational power posed the biggest obstacle. In early 2000, the Gene Expression Omnibus (GEO) project was initiated by the National Center for Biotechnology Information at the National Library of Medicine responding to the growing demand for an easily accessible public repository containing high-throughput gene expression data [12]. Additionally, other programs such as The Cancer Genome Atlas (TCGA) (https://www.cancer.gov/ccg/ accessed on 20 January 2024) run jointly by the National Cancer Institute (NCI) and the National Human Genome Research Institute and the Human Protein Atlas (https://www.proteinatlas.org/ accessed on 20 January 2024) further improved data availability, which combined with increased software and hardware capabilities allows for the development of potentially useful algorithms [13,14,15,16]. Therefore, in this manuscript, using a wide range of publicly available data, we selected potential FOLFOX resistance-related gene expression patterns that could be utilized as features in the decision-making algorithm that ingests problem-relevant information, to answer a question regarding the feasibility of FOLFOX treatment.

## 2. Results

### 2.1. Selection of Differently Expressed Genes (DEGs) in FOLFOX-Treated Responding and Non-Responding Patient Samples

**Figure 1:**
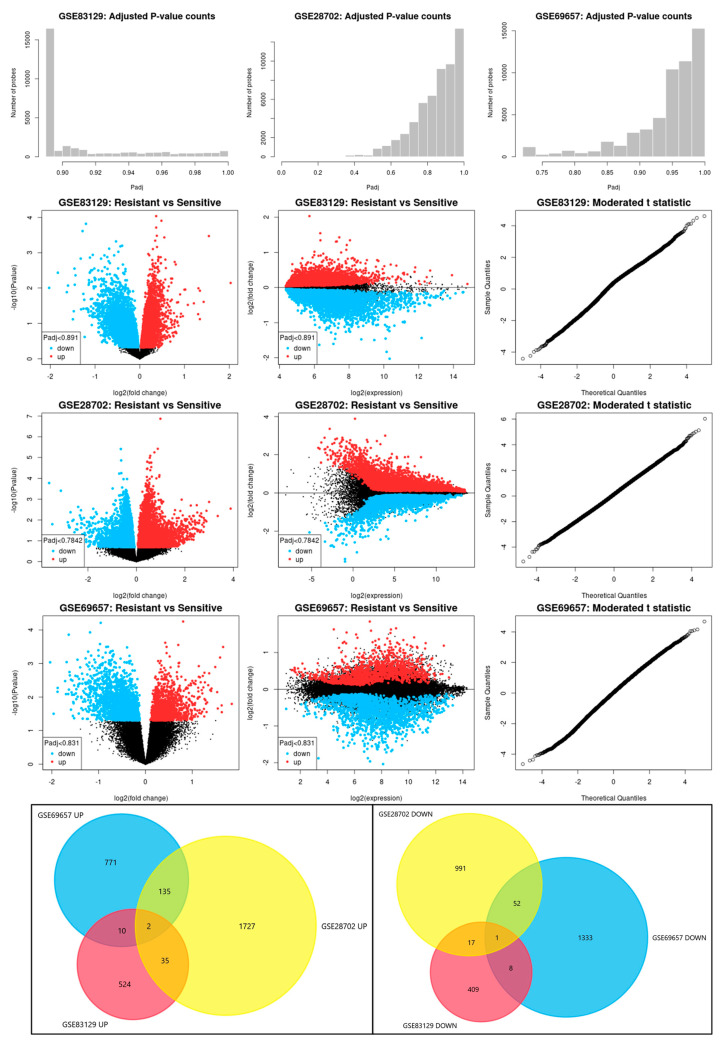
Differently expressed gene (DEG) distribution and selection using GEO GSE83129, GSE28702, and GSE69657 datasets. DEG distribution was analyzed using GEO2R and visualized by Volcano and Mean-difference plots. Data sets’ random probability distribution was analyzed by moderate t-stochastic plots. Commonly dysregulated genes for all 3 data sets were selected using Venn diagram and visualized by FunRich software (v3.1.3).

Transcriptomic data from CRC patients treated with neoadjuvant FOLFOX therapy were obtained from the GEO database (GSE83129, GSE28702, and GSE69657). Patients were stratified into two groups according to their objective response to chemotherapy using the Response Evaluation Criteria in Solid Tumours (RECIST), evaluated by computed tomography: FOLFOX non-responding—resistant (characterized by the best response being either ‘progressive disease’ or ‘stable disease’) and FOLFOX responding—sensitive (characterized by ‘partial response’ or ‘complete response’) [17,18,19]. In the GSE83129 data set, all non-tumor, adjacent samples were excluded from further analysis; thus, we obtained 9 resistant (FOLFOX non-responding) and 17 sensitive (FOLFOX responding) samples. In GSE28702, metastasis samples obtained from distant organs were excluded, and only primary samples were included in further analysis—25 resistant (FOLFOX non-responding) and 31 sensitive (FOLFOX responding) samples. In GSE69657, all samples were included—17 resistant (FOLFOX non-responding) and 13 sensitive (FOLFOX responding) samples. We did not observe any differently expressed genes with adjusted *p*-value (adj.*p*.Val): <0.05 in any of the tested data sets (Figure 1). Thus, we decided not to focus on one single dysregulated gene as a potential marker but rather on a combination of many discrete yet significant changes. We selected differently expressed genes (DEGs) that present raw *p*-value (*p*.Value) <0.05 and Moderated *t*-statistic value of 2 < (t) > −2 (with adj.*p*.Val for GSE83219 < 0.891, GSE28702 < 0.7842 and for GSE69657 < 0.831). Next, we excluded all doubled genes or unknown/unnamed sequences from all data sets. We obtained six sets of DEGs, two for each data set, composed of up- and downregulated genes in FOLFOX-resistant vs. sensitive CRC patient samples (Figure 1): GSE83219 UP *n* = 571, GSE83219 DOWN *n* = 435, GSE28702 UP *n* = 1899, GSE28702 DOWN *n* = 1061, GSE69657 UP *n* = 918, GSE69657 DOWN *n* = 1394. The majority of chosen genes were exclusively dysregulated in the single data set. Only two upregulated genes (*TMEM182* and *MCM9*) and one downregulated (*LRRFIP1*) were observed to be present in all corresponding sets.

### 2.2. Molecular Characteristic and Functional Enrichment Analysis of FOLFOX-Resistant CRC

**Figure 2:**
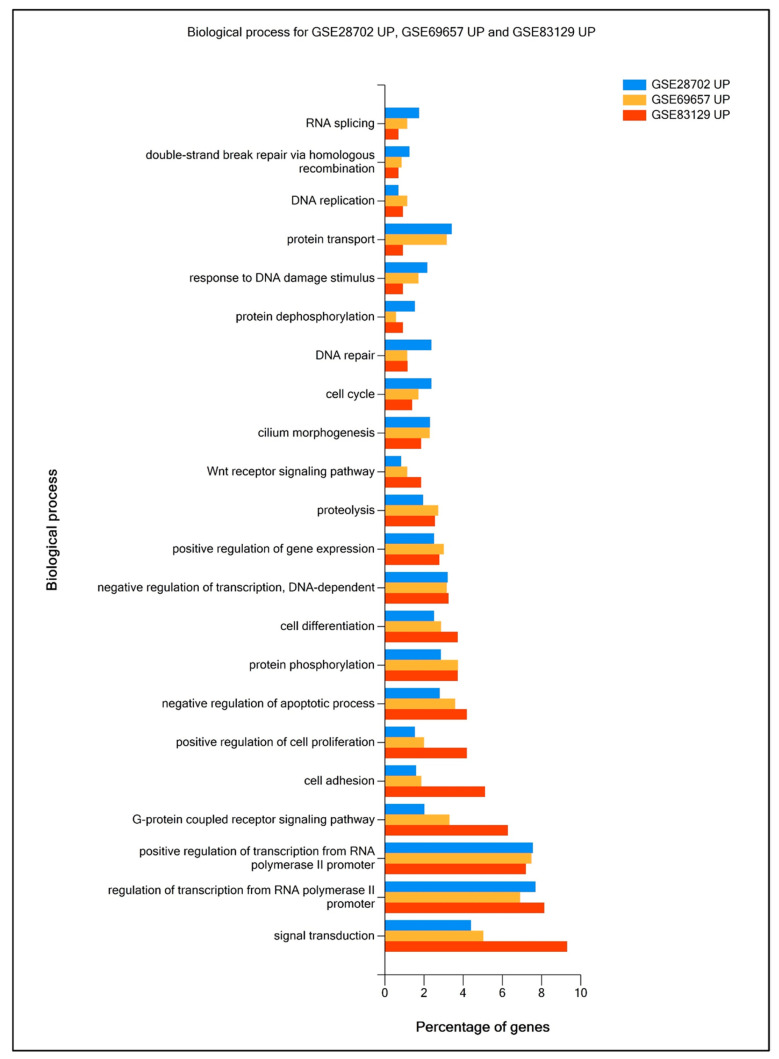
Comparative analysis of FOLFOX resistance-related upregulated gene functional enrichment. Percentage share of genes enriched in Biological process (Bp) were calculated and visualized using FunRich software (v3.1.3) supported by Gene Ontology (GO) database.

**Figure 3:**
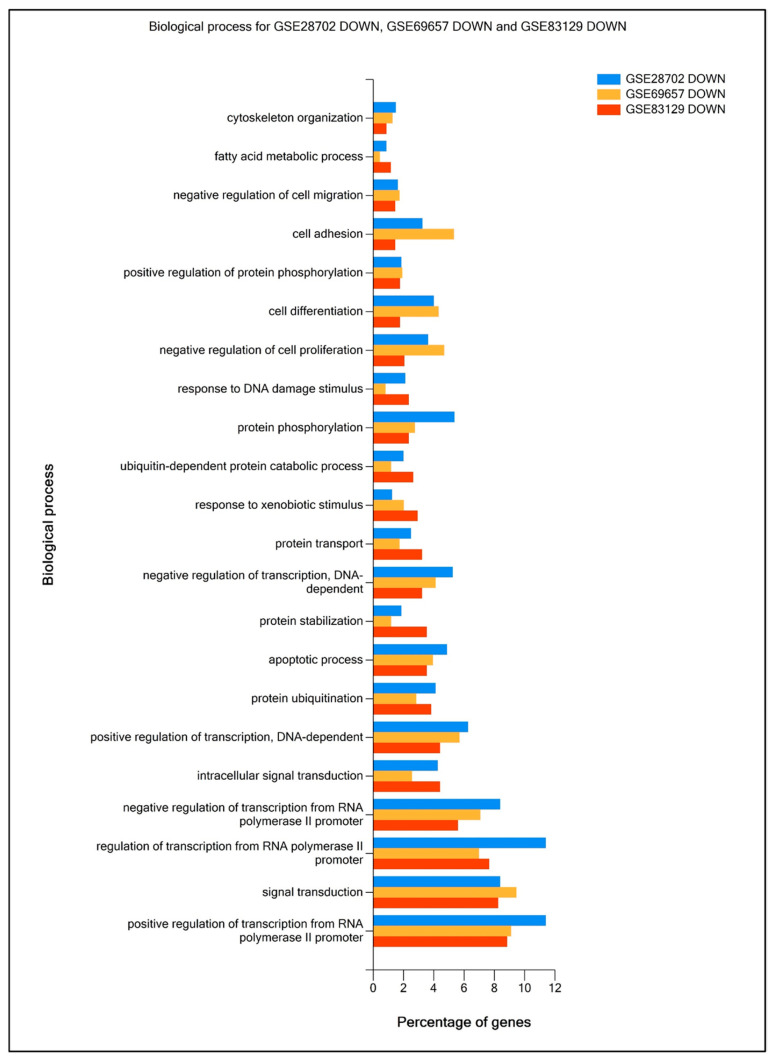
Comparative analysis of FOLFOX resistance-related downregulated genes’ functional enrichment. The percentage of genes enriched in Biological process (Bp) were calculated and visualized using FunRich software (v3.1.3) supported by the Gene Ontology (GO) database.

To verify functional enrichment differences between each data set, using FunRich 3.1.3 software and the Gene Ontology (GO) database, we compared top DEG enrichment in Biological process (Bp) (up-regulated genes presented in Figure 2 and downregulated genes—in Figure 3), Cellular components (Ccs), and Molecular functions (Mfs) (Supplementary Material) in analyzed data sets. Most of the proteins encoded by DEGs were involved in processes such as signal transduction, cell adhesion, transcription regulation, protein phosphorylation and ubiquitination, apoptosis, and cell proliferation. The percentage of top proteins encoded by DEGs enriched in Cc, Bp, and Mf in all data sets was similar; however, the most significant differences were observed between GSE29702 and GSE83219, especially in the case of nucleoplasm and plasma membrane enrichment (Cc), protein transport, positive regulation of cell proliferation, cell adhesion, G-protein coupled receptor signaling pathway and signal transduction (Bp) and DNA and RNA binding (Mf). In general, drug-resistant cancer cells are considered to retain the ability to proliferate under toxic conditions, as well as to present enhanced migratory properties [20,21,22]. The analyzed data prove that upregulated genes in FOLFOX-resistant patients’ samples, across all data sets, were enriched in DNA repair mechanisms, WNT signaling, positive regulation of cell proliferation, and negative regulation of apoptosis, whereas downregulated genes presented enrichment in the apoptotic process, negative regulation of migration and cell proliferation.

### 2.3. Selection of Potential FOLFOX Resistance Markers

**Figure 4:**
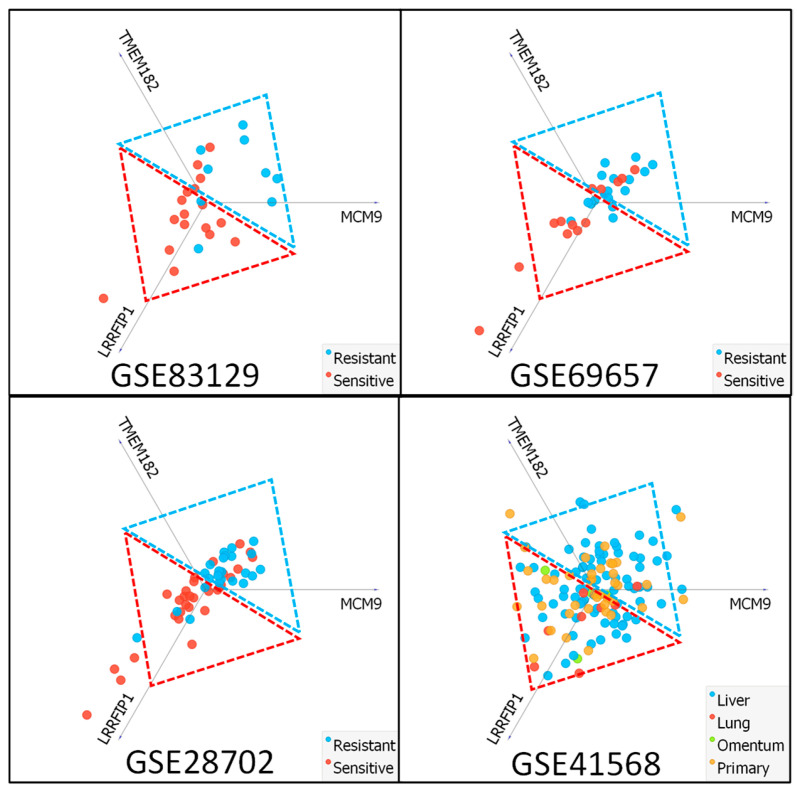
FOLFOX response discriminating abilities of *TMEM182*, MCM9, and *LRRFIP1* gene expression level. Visualization was performed using Orange data mining and linear projections. The Red dashed line triangle depicts predicted FOLFOX-sensitive instances of respective datasets, blue dashed line triangle depicts predicted FOLFOX-resistant instances. The actual FOLFOX response in samples from GSE83129, GSE28702, GSE69657, and GSE41568 datasets is presented as a red and blue color of depicted points. The GSE41568 data set presents predicted FOLFOX response of primary CRC, lung, liver, and omentum metastasis.

Comparing three data sets, only three genes were commonly dysregulated (in a statistically significant manner) for all sets. Two were upregulated (*TMEM182* and *MCM9*) and one was downregulated (*LRRFIP1*). Promoter DNA methylation is one of the most potent examples of gene expression regulation mechanisms, and aberrant methylation is found in almost every human cancer. Interestingly, using MEXPRESS (https://mexpress.be/ accessed on 20 January 2024) platform to evaluate the relationship between *TMEM182*, *MCM9*, and *LRRFIP1* expression and DNA methylation of their corresponding promotor region, we noticed that both upregulated genes present a negative correlation; thus, their expression is altered by DNA methylation. In contrast, the expression of *LRRFIP1* is DNA-methylation-independent (Supplementary Figure S2) [23]. Next, we verified whether the expression level of *TMEM182*, *MCM9*, and *LRRFIP1* may be used to predict FOLFOX resistance in GSE83219, GSE28702, and GSE69657. We used linear projections (Orange data mining software 3.31.1) to perform a visualization of labeled data. This function uses the linear transformation method that maps attribute values to points in a two-dimensional visualization space [24,25,26]. The obtained data prove that most resistant and sensitive samples are separated in a fairly correct, yet not perfect, manner (Table 1). This highlights the fact that the development of a more precise predictive algorithm is feasible. Furthermore, we analyzed potential FOLFOX resistance of primary and metastatic (lung, liver, and omentum) CRC samples (GSE41568) (Figure 4). Interestingly, the majority of lung (7 of 8) and omentum (3 of 4) metastasis samples presented FOLFOX sensitive phenotype. Moreover, the survival probability for CRC patients presenting the high and low expression of *TMEM182*, *MCM9*, and *LRRFIP1* was analyzed and visualized using the TIMER2.0 platform and TCGA database (Supplementary Figure S3) [14,27]. The 5-year survival rate (60 months) for patients with high expression of *TMEM182* was noticeably lower (57%) compared to patients with low expression (65%), similar to the MCM9 that presented, respectively, 57% and 63%. On the other hand, no significant changes in survival probability were observed for *LRRFIP1* (62% for high expression and 61% for low expression).

### 2.4. Selection of DEGs to Enhance the Correctness of FOLFOX Response Prediction

**Figure 5:**
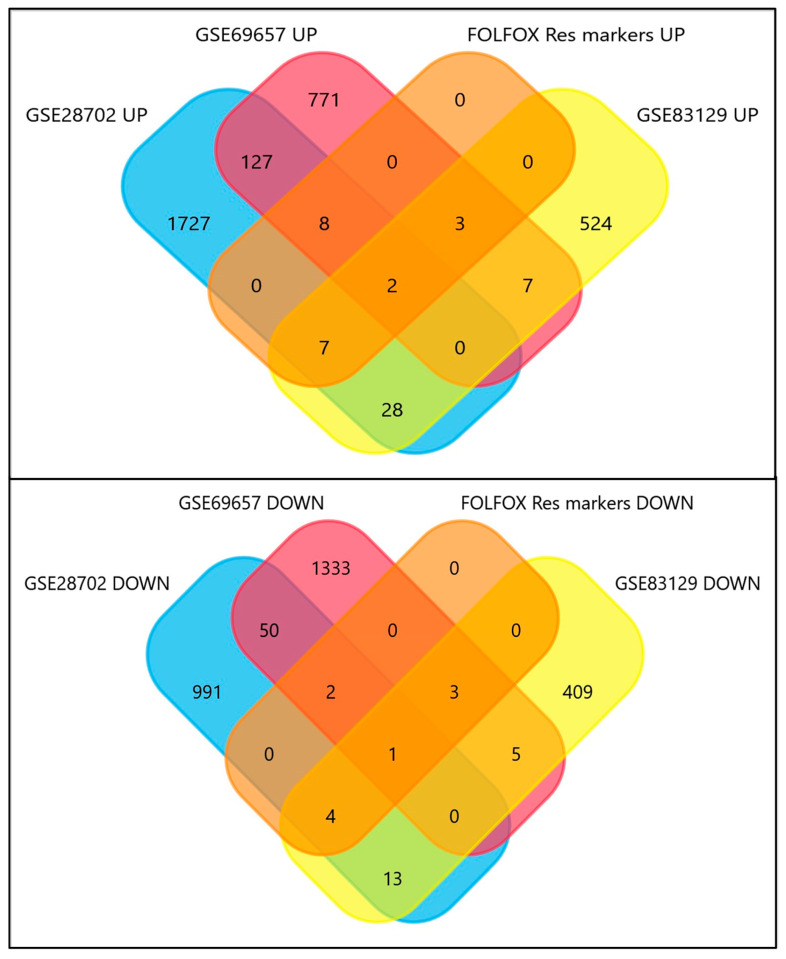
Visual depiction of FOLFOX resistance up- and downregulated genes that can be utilized as predictive features. Visualization was performed using FunRich software (v3.1.3).

To enhance the ability to separate between FOLFOX-resistant and sensitive samples, thus, between non-responding and responding patients, by creating a universal algorithm, we decided to increase the marker set. We verified the expression of all DEGs shared by each combination of two data sets in the remaining set. We excluded all genes that presented opposite regulation, t-value lower than 1 or higher than −1, and *p*-value less than 0.075. This approach allowed us to select potential markers—20 upregulated and 10 downregulated (Figure 5)—that present high *p*-value in at least two data sets and “acceptable” (*p* < 0.075) significance in the remaining one, and thus could be used to develop the predictive algorithm. Selected markers are presented in Table 2.

**Figure 6:**
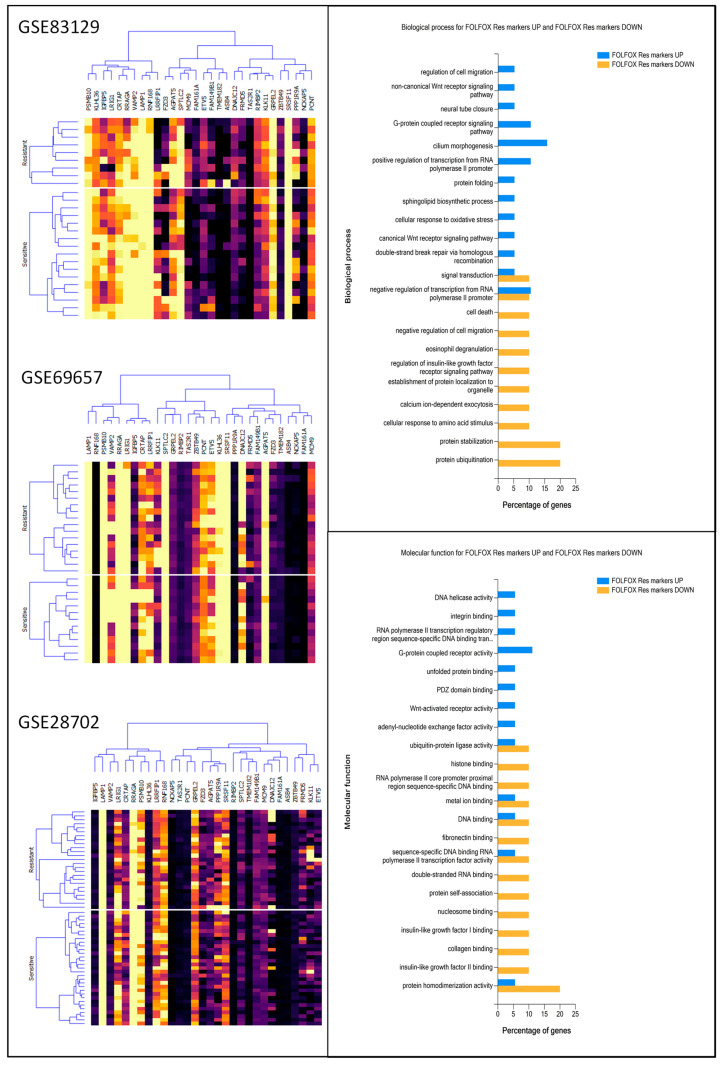
FOLFOX resistance marker characteristics. Hierarchical clustering analysis of chosen DEGs was analyzed and visualized using Orange data mining software for each data set. The percentage share of up- and downregulated genes functionally enriched in Biological process (Bp), and Molecular functions (Mf) were calculated and visualized using FunRich software (v3.1.3) supported by the Gene Ontology (GO) database.

Next, to verify chosen sets, we used Orange software to perform hierarchical clustering analysis (Figure 5) as we previously described [28]. All 10 downregulated genes, selected as potential markers, are located on one arm of the hierarchical clustering dendrogram (except for KLHL in GSE69657), whereas 20 upregulated are located on separate arm. This analysis strongly indicates that chosen DEGs could be utilized as a data set to create a universal prognostic algorithm. Additionally, to further analyze the selected DEGs, the Bp Cc, and Mf enrichment analysis was performed using FunRich 3.1.3 software and the Gene Ontology (GO) database (Figure 6, Supplementary Figure S4). The proteins encoded by the downregulated genes were predominantly involved in processes such as protein homodimerization activity, histone, DNA, metal ion, IGF, fibronectin and collagen binding and ubiquitin–protein ligase activity resulting in protein ubiquitination, protein stabilization, negative regulation of cell migration and IGF receptor signaling, and occurring in the cytosol, plasma membrane, nucleus, lysosome, endoplasmic reticulum, and extracellular space. On the other hand, proteins encoded by the selected up-regulated genes, in addition to the cytoplasm, and nucleus were enriched in the broader range of cellular components such as filopodium, chromosome, chromatin, nucleolus, mitochondria or adherent junctions, and involved in cellular processes such as WNT activity, DNA helicase activity, integrin binding, G-protein coupled receptor activity, PDZ domain binding, etc. resulting in double-strand brake repairs, protein folding, regulation of cell migration, canonical and non-canonical WNT signaling pathway and cellular response to oxidative stress.

### 2.5. Delivery of FOLFOX Resistance Predictive Algorithm

**Figure 7:**
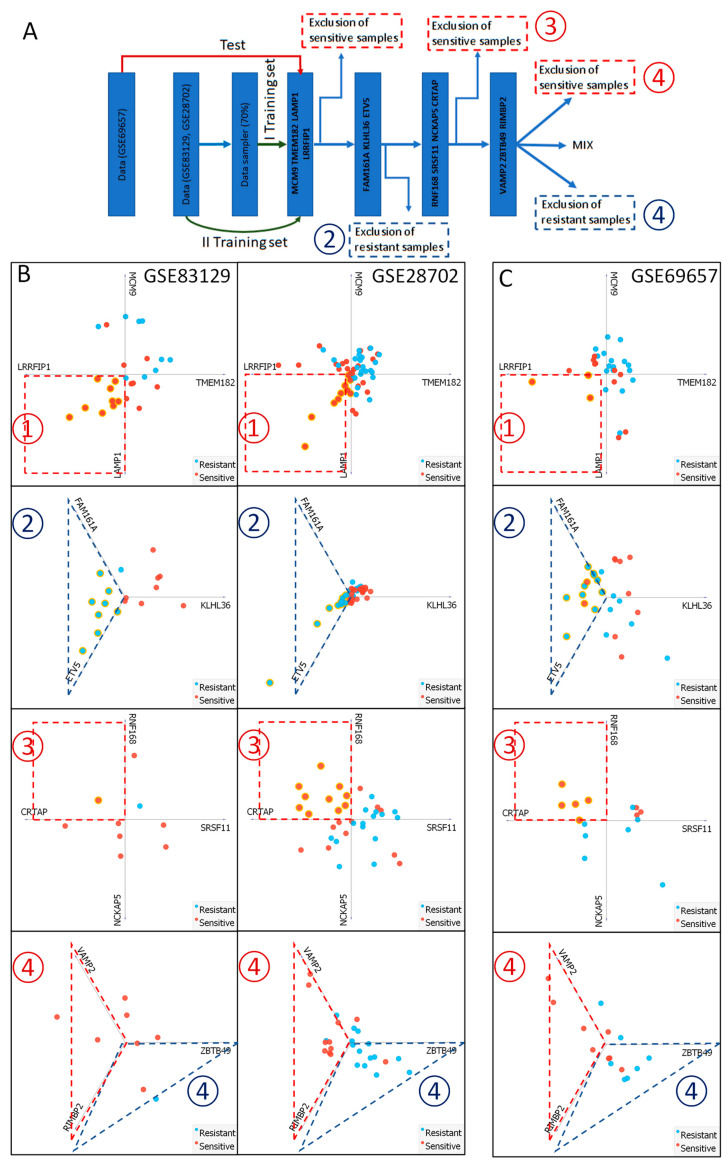
Step-by-step FOLFOX response predictive algorithm. (**A**) The block scheme of the step-by-step algorithm workflow. The first ① step uses the Cartesian coordinate system and expression level of *TMEM182*, *MCM9*, *LRRFIP1*, and *LAMP1* to eliminate only sensitive samples that are located in *LAMP1* and *LRRFIP1* quarter. Second strep ② uses *FAM161A*, *KLHL36*, and *ETV5* genes to eliminate resistant samples (located in the *FAM161A* and *ETV5* arm). The third step ③ again eliminates sensitive samples using *RNF168*, *SRSF11*, *NCKAP5*, and *CRTAP*, located in *CRTAP* and *RNF168* quarter. The final-forth step ④, with lower accuracy, separates sensitive, resistant, and mixed (hard to interpret) samples using *VAMP2*, *ZBTB49*, and *RIMBP2*. Sensitive samples are located on the *VAMP2* and *RIMBP2* arm, whereas resistant samples are on *RIMBP2* and *ZBTB49*. Lower panels present algorithm workflow using training sets GSE83129 and GSE28702 (**B**) and algorithms test group GSE69657 (**C**) with every step depicted as in (**A**). Visualization was performed using Orange data mining and linear projections. The dashed line depicts excluded clusters of homogenous response (red-sensitive, blue-resistant).

All three data sets differ from one another, containing different genetic and ethnic patient samples; thus, we attempted to create a universal algorithm that would predict FOLFOX resistance using mRNA levels of selected genes. Methods based on mathematical modeling, supported by machine learning, that correlate survival probability with the expression of specific genes are often used to determine and validate several risk factor markers to predict cancer progression or/and therapy outcome. Most often, they use algorithms based on receiver operating characteristic (ROC) curves, Kaplan–Meier estimation of progression-free survival, cancer-specific survival, and overall survival (OS) rate [16,29]. However, the majority of such algorithms are complicated and challenging to reproduce during clinical decision-making by oncologists. Therefore, we decided to simplify by not over-simplifying this complexity and apply a stepwise modified decision tree-based algorithm using radial data visualization (via linear projection and open-source data mining software Orange). In this approach, each step eliminates homogeneous samples only (according to a chosen set of features) from the total population, and subsequently reduces difficult to interpret samples to a minimum through multiple rounds of elimination. Our analysis show that the GSE69657 and GSE28702 data sets were more similar to each other than to GSE83129, sharing 135 up-regulated and 52 down-regulated genes. They also subjectively visualize similar dispersion of samples in linear projections based on the mRNA expression of *TMEM182*, *MCM9*, and *LRRFIP1.* Thus, to develop the FOLFOX resistance predictive algorithm, we used GSE83129 and GSE28702 as training sets and GSE69657 as test sets (Figure 7). On every step, we used data-driven linear transformations that focus on visualized multivariate data in a low-dimensional space (visualization space) that is optimal for easy graphical interpretation [24,25]. We mapped attribute values (features) based on the expression level of 3 or 4 genes to points in a two-dimensional visualization space using a simple linear projection widget and Orange data mining software to eliminate a portion of homogenous samples presenting either FOLFOX sensitive or FOLFOX-resistant phenotype. Features were selected from our 30 potential markers, and each step was trained using the circular placement of suggested features, first by randomly assigning 70% of each training set and then verifying them using all training data. This approach by using the geometric visualization method, relies only on the values of attributes (gene expression levels) to influence the position of the points in a low-dimensional space [30]. The first step uses the Cartesian coordinate system and expression level of our 3 marker genes, *TMEM182*, *MCM9*, and *LRRFIP1*, additionally supported by *LAMP1* to eliminate only sensitive samples that are located in *LAMP1* and *LRRFIP1* quarter. Next, the remaining samples were separated using 3 gene sets: *FAM161A*, *KLHL36*, and *ETV5* to eliminate resistant samples (located in the *FAM161A* and *ETV5* arm). The third step again eliminates sensitive samples from 4 genes, Cartesian coordinate system *RNF168*, *SRSF11*, *NCKAP5*, and *CRTAP*, located in *CRTAP* and *RNF168* quarter. The final step, with lower accuracy, separates sensitive, resistant, and mixed (hard to interpret) samples using *VAMP2*, *ZBTB49*, and *RIMBP2*. Sensitive samples are located on the *VAMP2* and *RIMBP2* arm, whereas resistant samples are on *RIMBP2* and *ZBTB49*. The obtained algorithm was verified by the GSE69657 test set, which proves that the proposed algorithm sufficiently discriminates (in a subjective manner) FOLFOX resistant and sensitive samples with approximately 18.5% of samples presenting incorrect (falsely resistant) categorization, simultaneously showing higher than 90% accuracy in both training sets. Significantly, none of the FOLFOX-resistant samples of the test set were incorrectly classified as sensitive. Additionally, expression levels of chosen genes (presented in Supplementary Figure S5A–C), used as distinctive attributes in our procedure, were analyzed using the Pearson correlation matrix (Supplementary Figure S6A–C) as we previously described [28]. The expression level of the majority of up and down-regulated genes selected as potential markers positively correlates within their distinguished group, with simultaneous negative correlation to the members of the opposite group in the GSE83129 and GSE28702 training sets and GSE69657 test set.

### 2.6. Algorithm Verification

**Figure 8:**
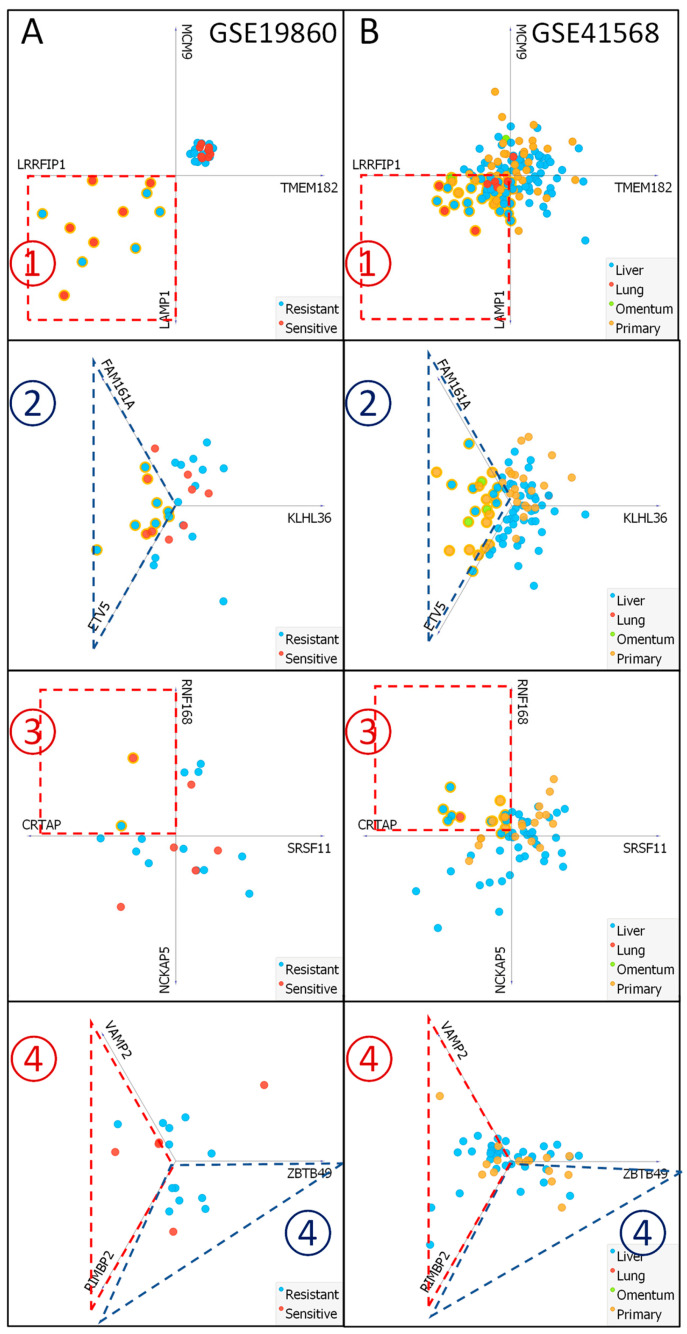
FOLFOX response discriminating abilities of the proposed step-by-step algorithm, based on the expression level of chosen DEGs using the GSE19860 (**A**) and GSE41568 (**B**) GEO datasets. Visualization was performed using Orange data mining and linear projections. The dashed line depicts excluded clusters of homogenous response (red-sensitive, blue-resistant). Each step is depicted according to Figure 7A as ①–④.

Finally, to analyze the ability of our method to predict therapy outcomes regardless of the data value presentation system, we ran through the algorithm two new data sets that were not used to identify or select algorithms attributes. The GSE19860 data set consists of samples from metastatic or recurrent CRC patients who received FOLFOX treatment and presented sensitive or resistant phenotypes (responding and non-responding), and the GSE41568 data set consists of primary and metastasis CRC patient samples that received at least 3 months of oxaliplatin-based therapy and underwent resection of their cancer within 4–6 weeks after their last dose with no information regarding the occurrence of FOLFOX (or oxaliplatin) resistance (Figure 8) [31]. Importantly, the GSE19860 data set comprises a mixed value data presentation system (MAS5-calculated Signal intensity and PerGene normalized signal intensity). This fact was purposely used to verify algorithm versatility and its independence of data standards. A verification run performed using GSE19860 data proved that identification of FOLFOX resistant samples is more correct than identification of FOLFOX sensitive ones (in mixed data presentation environment). Seventeen samples were classified (by algorithm) as FOLFOX resistant, with 14 being actually FOLFOX non-responder, according to clinical data description. In comparison, FOLFOX-sensitive group with 17 samples was composed of 9 actual FOLFOX responders and 8 FOLFOX non-responders. Additionally, the hard-to-interpret group was mainly composed of FOLFOX resistant samples (4 non-responders and 1 responder). During the verification run performed using GSE41568 that provides no information on FOLFOX response, a heterogeneous group of primary and metastatic CRC samples was classified as FOLFOX sensitive and resistant. Most of the primary CRCs were found to present a resistant phenotype. Additionally, 11 patients with liver metastasis received neoadjuvant chemotherapy prior to the 3 months of Oxaliplatin treatment, 7-FOLFOX (including one receiving FOLFOX + bevacizumab), and 4 received XELOX + bevacizumab. Interestingly, 3 samples were categorized as resistant by our algorithm, 2 of which were treated with a combination of XELOX + bevacizumab (CRC098D and CRC174B) and one with FOLFOX (CRC159). However, the most crucial observation is that our algorithm classifies all CRC lung metastasis samples as sensitive, potentially responding to the FOLFOX treatment.

Additionally, using GSE44861 data set, we have compared selected genes expression levels between CRC samples and adjacent tissue (Supplementary Figure S7). *CRTAP*, *ETV5*, and *LAMP1* present significantly higher expression level in CRC samples whereas, *LRRFIP1*, *KLHL36*, *VAMP2*, and *RIMBP2* in noncancerous adjacent tissue.

## 3. Discussion

Colorectal cancer as the second leading cause of cancer death, presents an increasing incidence, especially in developing and developed countries [32]. CRC metastasis is predominantly observed in the liver, 25–30% at the initial stage, and approximately 50% will develop during the treatment, followed by the lung metastasis, 10–15% [33,34,35]. A total of 4–10% of CRC metastasis is observed in the abdominal wall, greater omentum and peritoneum [36]. Currently, the most effective treatment for CRC is surgical excision of the early primary tumor [1]. However, approximately 30% of stage II and 50–60% of stage III CRC patients who have undergone tumor resection will finally develop recurrence within 5 years [37]. Thus, chemotherapy remains one of the most important anti-cancer strategies. Unfortunately, upon chemotherapy application, CRC cells often develop resistance to its components [6]. This complicated multifactorial network of often complementary resistant related mechanisms is mainly responsible for the failure of chemotherapy, highlighting the necessity of developing a precise algorithm that predicts patients’ response to given therapy [21,38,39].

In the early 90s, a cognitive scheme for AI decision-making protocol was proposed, stating that the diagnostic task proceeds through three different phases: hypotheses generation followed by hypotheses testing, and finally hypotheses closure (verification) [40]. Correct predictive algorithms imply a cognitive task that uses pre-trained networks, with internal parameters that vary according to a set of fixed rules during a training cycle, that are internally corrected each time a given pattern is erroneously classified [10,11,16,41]. Hypotheses testing is achieved by a deductive inference, followed by an eliminative induction, performed using an already trained and tested scheme. Then, hypotheses verification matches the consequences of the generated hypotheses against the patient’s characteristics or preferences under the utility criterion [40]. Therefore, the most important factor is correct algorithm construction based on proper data selection. However, the development of a hypothetical model presenting clinical useability remains challenging due to many factors, such as lack of precise pharmacogenomic data, cancers heterogeneity, model overcomplication, multicollinearity, and overfitting of data, and many others [16]. For a given treatment, the clinical response and the treatment efficacy are determined by basic measurements, such as the quantification of tumor shrinkage and OS, that were not designed to reveal the underlying complexity of the response based on various resistance levels in different cells in a tumor [42]. Furthermore, the application of these methods is limited in many cancer studies, as the corresponding time-to-event outcomes of interest are invariably subject to interval-censoring mechanisms by clinical practice or study design [29]. Nevertheless, no better tools are currently available to collect vast amounts of clinical data regarding treatment response.

In the current manuscript, using three independent GEO database data sets, we have selected three genes, *TMEM182*, *MCM9*, and *LRRFIP1*, differently expressed in all three data sets. *TMEM182*, *MCM9* were upregulated in FOLFOX-resistant and *LRRFIP1* was downregulated in FOLFOX non-responding samples. The expression level of selected genes could be utilized to predict CRC patient’s response to the FOLFOX treatment, discriminating resistant (non-responding) and sensitive (responding) CRC patients in a satisfactory (yet not perfect) manner. From those three selected genes, only *MCM9* and *LRRFIP1* present some connotation with CRC. MCM9 protein, whose physiological functions are based on MCM9/MCM8 complex that participates in a helicase hexameric complex involved in homologous recombination during the repair of double-strand breaks, is potentially responsible for hereditary CRC [43]. LRRFIP1, that regulates a wide range of biological processes, such as immune response, cytoskeletal remodeling, signal transduction, and transcriptional regulations of genes (including regulation of EMT) in the case of CRC, via regulation of RhoA-induced cell adhesion, migration, and invasion, plays an important role in metastasis to the liver [44,45,46]. The transmembrane (TMEM) protein family includes proteins with mostly unknown functions; however, several in vitro and in vivo studies proved that protein encoded by *TMEM182* plays inhibitory function in skeletal muscle development, growth, and regeneration [47]. Contrary to our method, markers typically associated with FOLFOX resistance, such as ABCC2, have no clinical association with early relapse of CRC in patients receiving FOLFOX and cannot be used to create a reliable model [48]. Importantly, chemotherapy resistance, presented by cancer cells, is a complicated net of different mechanisms and differs from patient to patient [21]. Thus, to further improve predictive ability, we have expanded the potential marker list up to 30 genes that are not directly involved in CRC detoxication or repairs of oxaliplatin-derived DNA damage, using similar verification methods based on the integrated bioinformatics, as presented in our previous paper regarding FOLFIRI resistance markers in CRC [28]. Next, a simple algorithm, based on a modified decision tree, that step-by-step eliminates samples presenting either FOLFOX responding or non-responding phenotype with over 80% correctness was delivered. Unfortunately, the majority of data were collected in Asian or Caucasian populations thus may not perfectly represent other (such as African and African American) populations. Despite the fact that representatives of the Black population (in the US) have higher CRC incidence rates and lower survival compared to other races, their oncology trial participation and patient data availability are lacking [49,50]. Genes, which expression level were used as the attributes, such as *FAM161A*, *KLHL36*, *ETV5*, and *CRTAP* have no known connotation with CRC. *LAMP1* gene is the most prominently expressed in microsatellite instability (MSI)-depleted CRC fraction, and has not yet been related to the FOLFOX resistance or sensitivity [51]. High expression of *ETV5*, similar to *ETV1*, indicates advanced stage and poor prognosis of CRC patients, mainly due to correlation with increasing immune infiltration with EMT-inducing M2 macrophages and cancer-associated fibroblasts (CAFs) [52]. RNF168, a DNA damage response (DDR) factor regulated by one of the EMT triggering transcription factors—ZEB1—leads to chromosome stability and promotes resistance to 5-Fluorouracil, but not to oxaliplatin in CRC [53]. Due to the dysregulation of alternative splicing, the expression of splicing factor SRSF11 is associated with the acquisition of metastatic potential and poor survival of CRC patients [54]. NCKAP5 was recently identified as a prognostic biomarker in NSCLC. Its expression correlates with immune cell infiltration, and overall poor prognosis in this type of cancer; however, no data yet are available, regarding its involvement in CRC chemoresistance [55]. VAMP2 is involved in exosome secretion via the multivesicular body (MVB) membrane fusion, and thus, it is directly involved in the preparation of premetastatic niche and CRC progression, with no direct impact on tumor chemoresistance [56]. ZBTB49 is highly expressed in normal epithelial cells, as was shown by the immunohistological analysis but is repressed in colon, lung, and skin tumor tissues. However, its expression is induced by p53 in response to DNA damage, leading to cancer cell cycle arrest [57]. RIMBP2 mRNA expression level correlates with the worst prognosis for lung squamous carcinoma patients and was found to be among 8 differentially expressed genes in lenvatinib-treated hepatocellular carcinoma [58,59]. Among the various supervised learning algorithms, the decision tree is a relatively popular predictive modeling algorithm, used to classify simple data. According to a test rule, a decision tree takes data in the root node and keeps growing until it reaches a decision. Each internal node, representing different features, breaks the data into a small subset until it meets a particular condition. Each step can be easily understood, interpreted, and visualized [16]. Our algorithm was based on a few simple assumptions: (a) overall design and data interpretation must be simple and intuitive, (b) each step must eliminate a homogenous fraction of samples presenting specific features, (c) the procedure requires minimal data preparation with no specially dedicated sophisticated software or hardware, (d) it must be data distribution independent, and finally (e) relatively fast to train and utilize. Thus, using linear projection of different gene expression levels, we have developed a simple, yet precise systematic procedure that was trained and tested on both CRC patient samples and in vitro delivered oxaliplatin-resistant variants of CRC cell lines. Linear projections based on radial visualization use the geometric visualization method where given samples are visualized as points in a low-dimensional space, and the values of attributes (gene expression levels) influence only the position of the points [30]. This method was explained by Leban et al. [24] using a simple physical analogy with multiple springs attached with one end to the attribute (in this instance, the chosen gene) and with the other end to the data point inside the visualization space. The stiffness of each spring in terms of Hooke’s law is determined by the corresponding attribute value (in this case, chosen gene expression level); thus, the greater the attribute value, the greater the stiffness, and the data point is drawn closer to “stronger” attributes. In the aftermath, the data point is placed where the sum of all spring forces equals 0 [24]. This simple, yet not over-simplified, approach is distribution-independent and can be applied to data obtained using different methods and require almost no data preparation. This fact was further tested and proven using GSE19860 data set, as it is composed of mixed value data presentation system containing both raw signal intensity and normalized signal intensity.

Our procedure allowed us to analyze the potential FOLFOX response of primary and metastatic CRC samples (GSE41568). The obtained data prove that despite the previous 3 months of FOLFOX treatment, CRC lung metastates remain sensitive, suggesting that FOLFOX administration prior to the surgical exertion should be considered therapeutically beneficial. A recent meta-analysis based on a total of 1936 patients from eight independent studies proved that peri-operative and adjuvant chemotherapy (FOLFOX) given to patients with resectable pulmonary CRC metastases increases their survival rate [2].

Unfortunately, the biggest drawback of every developed predictive model is the consistency and correctness of data description. We have based our method on available clinical descriptions of patients’ responses to FOLFOX treatment obtained by computer tomography according to RECIST guidelines. Even though, currently, the majority of clinical trials continue to use RECIST, the RECIST Working Group constantly reviews the criteria and updates them periodically in response to both therapeutic and imaging technology advances. Nevertheless, this fact allows for particular misinterpretation of samples, those belonging to either the ‘Stable disease’ or ‘Partial response’ group [60]. Furthermore, to create our algorithm, we have excluded samples from patients who were treated with a combination of FOLFOX and other therapy forms, thus an additional limitation resulting from possible, not fully correct, sample description might have caused some co-treated patients’ samples to be used. Nevertheless, it would not significantly impact overall results.

## 4. Material and Methods

### 4.1. Microarray Data Processing and Analysis

Gene expression profiles with accession numbers: GSE83129, GSE28702, and GSE69657 were downloaded from The Gene Expression Omnibus (GEO) database (http://www.ncbi.nlm.nih.gov/geo/ accessed on 20 January 2024) and analyzed similarly to our previous work using integrated bioinformatics [28].

GSE83129—this data set is composed of n = 36 colorectal cancer patient samples (26 adenocarcinoma samples and 10 normal adjacent tissue samples) obtained by laser-microdissection from biopsies of first-line oxaliplatin and 5-FU (FOLFOX) treated metastatic colorectal cancer patients from the Departments of Odense University Hospital, Odense, Denmark and Aarhus University Hospital, Aarhus, Denmark [18]. Gene expression profiles were obtained using GPL6244 Affymetrix Human Gene 1.0 ST Array.

GSE28702—this data set is composed of n = 83 unresectable colorectal cancer patient samples (42 FOLFOX responders and 41 FOLFOX non-responders) obtained from the Teikyo University Hospital at Mizonokuchi and Gifu University Hospital. Patients did not receive any chemotherapy or radiotherapy before the study, where they were subjected to the FOLFOX regime [17]. Gene expression profiles were obtained using GPL570 Affymetrix Human Genome U133 Plus 2.0 Array (Thermo Fisher Scientific, Waltham, MA, USA).

GSE69657—this data set is composed of n = 30 post-chemotherapy colorectal cancer patient samples (13 FOLFOX responders and 17 FOLFOX non-responders) obtained from Fujian Medical University Union Hospital [19,61]. Gene expression profiles were obtained using GPL570 Affymetrix Human Genome U133 Plus 2.0 Array (Thermo Fisher Scientific, Waltham, MA, USA).

GSE19860—this data set is composed of n = 40 CRC patients who underwent surgery for primary lesions. All patients had metastatic or recurrent CRC and received FOLFOX therapy in the Department of Surgical Oncology, University of Tokyo. Responders and non-responders were determined based on the best-observed response at the end of the first-line treatment, resulting in n = 15 FOLFOX responders (sensitive) and 25 FOLFOX non-responders (resistant). Gene expression profiles were obtained using Affymetrix Human Genome GeneChip arrays U133 (Thermo Fisher Scientific, Waltham, MA, USA).

GSE41568—this data set is composed of n = 133 freshly frozen primary and metastatic colorectal cancer patients’ tissue samples obtained from Duke University, Durham, NC, USA. All patients received at least 3 months of Oxaliplatin-based therapy and underwent resection of their cancer within 4–6 weeks after their last dose [31]. Gene expression profiles were obtained using the GPL570 platform Affymetrix Human Genome U133 Plus 2.0 Array (Thermo Fisher Scientific, Waltham, MA, USA).

GSE44861—this data set is composed of n = 111 from freshly frozen colon tissue samples from CRC tumors and adjacent noncancerous tissues obtained from the Laboratory of Human Carcinogenesis, Bethesda, MD, USA [62]. Gene expression profiles were obtained using the GPL3921 platform Affymetrix HT Human Genome U133A Array (Thermo Fisher Scientific, Waltham, MA, USA).

All data were processed using the GEO2R online analytical tool, which uses the R language (#Version info: R 4.2.2, Biobase 2.58.0, GEOquery 2.66.0, limma 3.54.0) [63]. Linear projections of gene mRNA level, principal component analysis (PCA), and t-distributed Stochastic Neighbor Embedding (t-SNE) were performed using Orange software as previously presented by us [28].

### 4.2. Enrichment Analysis

Functional enrichment software tool FunRich (v3.1.3) (http://www.funrich.org/) supported by Gene Ontology (GO) (http://geneontology.org/) (accessed on 20 January 2024) database was used to compare, analyze, and visualize the Cellular components (Cc), Biological process (Bp) and Molecular functions (Mf) differences associated with the proteins encoded by differently expressed genes (DEGs) in FOLFOX-resistant and sensitive CRC patient samples.

### 4.3. Hierarchical Clustering Analysis

After extracting the expression values from the gene expression profiles, a bidirectional hierarchical clustering heatmap of differently expressed genes (DEGs) was calculated and visualized using the Orange open-source machine learning and data visualization platform as previously described by us [28].

### 4.4. Pearson Correlation Analysis

Pearson correlation analysis of chosen DEGs was calculated and visualized using JASP 0.14.1.0 software (https://jasp-stats.org/ accessed on 20 January 2024) with color depiction of “r” value and significance * *p* < 0.05, ** *p* < 0.005, and *** *p* < 0.001 as previously described by us [28].

### 4.5. Gene Methylation Analysis

Analysis and visualization of methylation involvement in the regulation of chosen gene expression were performed using the MEXPRESS (https://mexpress.be/ accessed on 20 January 2024) platform supported by the TCGA database [23]. The expression of chosen genes was analyzed in colon adenocarcinoma samples deposited in the TCGA database; next, it was correlated with the methylation of CpG island in the promoter region.

### 4.6. Survival Probability Analysis

The survival rate for patients presenting the high and low expression of chosen DEGs was analyzed in CRC patients using TCGA data, the Human Protein Atlas (www.proteinatlas.org accessed on 20 January 2024) “pathology” section [15,27] and TIMER2.0 platform (http://timer.cistrome.org/ accessed on 20 January 2024) [64]. Presented data used the best expression cut-off suggested by HPA and were composed of, respectively, *TMEM182*: n = 470 “low” expression patients and n = 1127 “high” expression patients, *MCM9:* n = 401 and n = 196, *LRRFIP1:* n = 410 and n = 187.

## 5. Conclusions

The step-by-step eliminative procedure, based on modified radial visualization of chosen gene expression level (as attributes) proved to be a capable and easy-to-use tool, to predict potential patients’ response to FOLFOX treatment. Drug resistance is a complicated network of many mechanisms, generating highly heterogenous responses in CRC patients. Thus, we have minimalized incorrect data separation by focusing on homogenous data separation in several successive steps, providing discreet step-by-step selection of resistant and sensitive patients that presents various approaches to FOLFOX resistance (different gene expression pattern). Potential FOLFOX resistance markers (*TMEM182*, *MCM9*, *LRRFIP1*, *LAMP1*, *FAM161A*, *KLHL36*, *ETV5*, *RNF168*, *SRSF11*, *NCKAP5*, *CRTAP*, *VAMP2*, *ZBTB49* and *RIMBP2*) were selected and validated using GSE83129, GSE28702, GSE69657, GSE19860 and GSE41568 data sets. As our algorithm was based on data obtained by commercially available microarrays, we reason that the creation of a simple test based on such existing and already developed components is possible and easy to produce; thus, our approach can provide new insight into clinical decision-making regarding accurate therapy scheme administration. The biggest weakness of proposed predictive procedure is the selection of the correct markers. They were based on mainly Asian and Caucasian patients’ data description, and obtained by computer tomography according to RECIST guidelines. This fact employs human error into algorithm development. Thus, further verification to increase our procedure’s application and reproducibility are needed.

## Figures and Tables

**Table 1:** FOLFOX response discriminating abilities based on the linear projection of *TMEM182*, *MCM9*, and *LRRFIP1* gene expression level.

FOLFOX Response:	GSE83219		GSE69657		GSE28702	
	Selected	Correct	Selected	Correct	Selected	Correct
Sensitive	15	13 (86%)	12	8 (67%)	23	20 (87%)
Resistant	11	7 (63%)	18	13 (72%)	33	22 (67%)

**Table 2:** Selected markers of FOLFOX resistance in CRC.

No.	Gene	Protein
UP:		
1	*TMEM182*	Transmembrane protein 182
2	*MCM9*	Minichromosome maintenance 9 homologous recombination repair factor
3	*PCNT*	Pericentrin
4	*KLK11*	Kallikrein-11
5	*TAS2R1*	Taste receptor type 2 member 1
6	*DNAJC12*	DnaJ heat shock protein family member C12
7	*ZBTB49*	Zinc finger and BTB domain-containing protein 49
8	*SRSF11*	Serine/arginine-rich splicing factor 11
9	*FZD3*	Frizzled class receptor 3
10	*AGPAT5*	1-acyl-sn-glycerol-3-phosphate acyltransferase epsilon
11	*FAM149B1*	Protein FAM149B1, family with sequence similarity 149 member B1
12	*FRMD5*	FERM domain-containing protein 5
13	*ETV5*	ETS translocation variant 5
14	*GRPEL2*	GrpE protein homolog 2
15	*ASB4*	Ankyrin repeat and SOCS box protein 4
16	*FAM161A*	Family with sequence similarity 161 members a
17	*RIMBP2*	RIMS-binding protein 2
18	*PPP1R9A*	Neurabin-1
19	*SPTLC2*	Serine palmitoyltransferase 2
20	*NCKAP5*	NCK-associated protein 5
DOWN:		
21	*LRRFIP1*	Leucine-rich repeat flightless-interacting protein 1
22	*LAMP1*	Lysosome-associated membrane glycoprotein 1
23	*KLHL36*	Kelch-like protein 36
24	*PSMB10*	Proteasome subunit beta type-10
25	*RRAGA*	Ras-related GTP-binding protein A
26	*CRTAP*	Cartilage-associated protein
27	*LRIG1*	Leucine-rich repeats and immunoglobulin-like domains protein 1
28	*RNF168*	E3 ubiquitin-protein ligase RNF168
29	*VAMP2*	Vesicle-associated membrane protein 2
30	*IGFBP5*	Insulin-like growth factor-binding protein 5

## Data Availability

The data presented in this study are available in The Gene Expression Omnibus at http://www.ncbi.nlm.nih.gov/geo/ reference number GSE83129; GSE28702; GSE69657; GSE19860; GSE41568.

## Supplementary Materials

The following supporting information can be downloaded at: https://www.mdpi.com/article/10.3390/ijms252212149/s1.
