# Supplementary material for: Radial Data Visualization-Based Step-by-Step Eliminative Algorithm to Predict Colorectal Cancer Patients’ Response to FOLFOX Therapy
Source: Int J Mol Sci. 2024 Nov 12;25(22):12149. doi: 10.3390/ijms252212149 (PMC11595261; doi:10.3390/ijms252212149)
Supplement: Supplementary file 1 [file ijms-25-12149-s001.zip › ijms-3237165-supplementary.pdf]

Supplementary files

Supplementary S1A

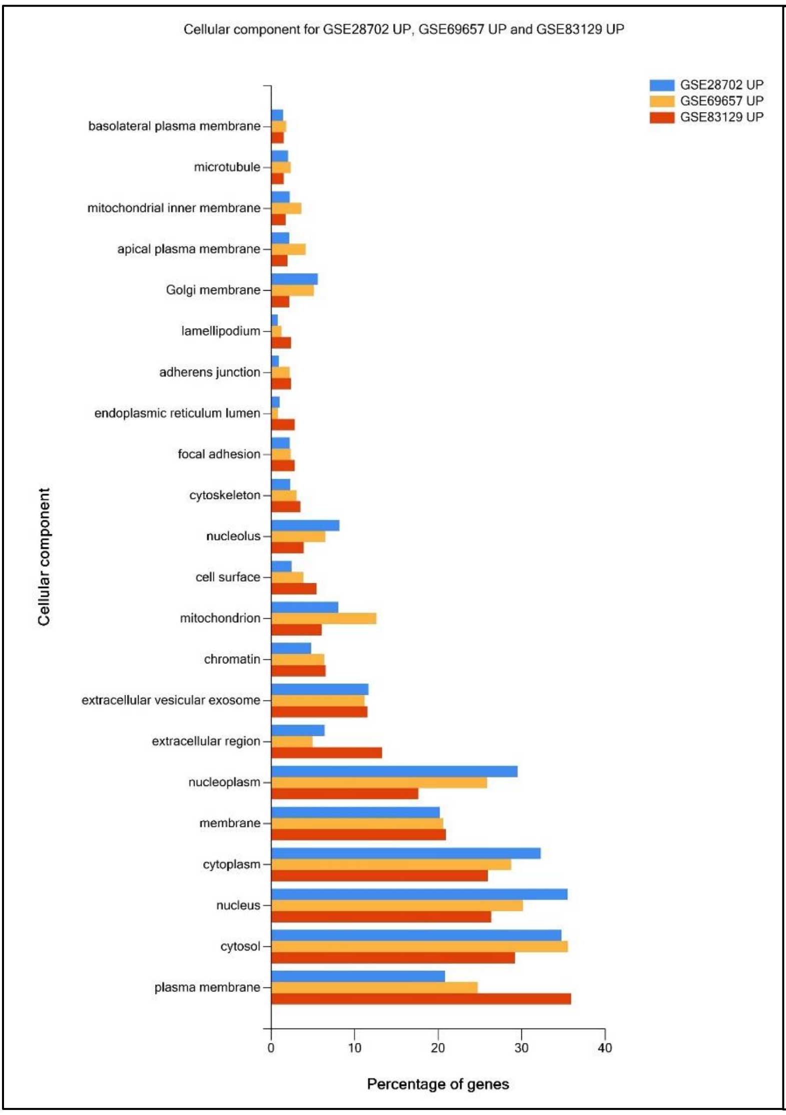

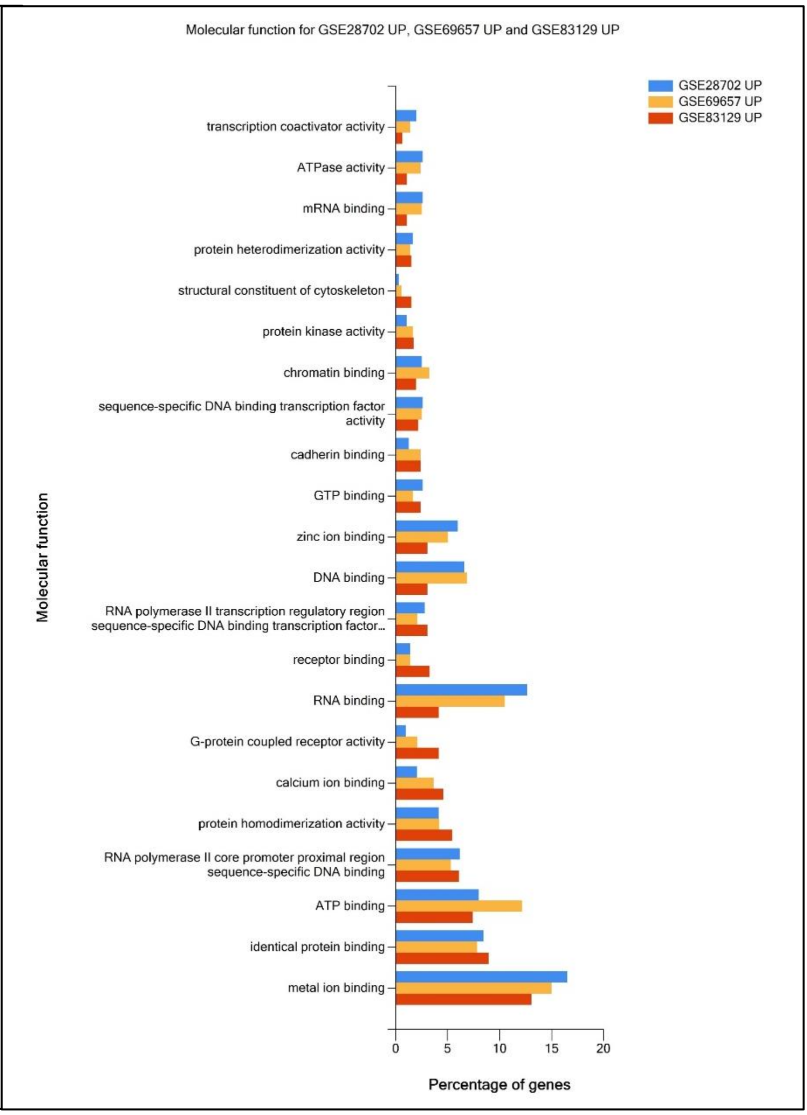

Supplementary S1B

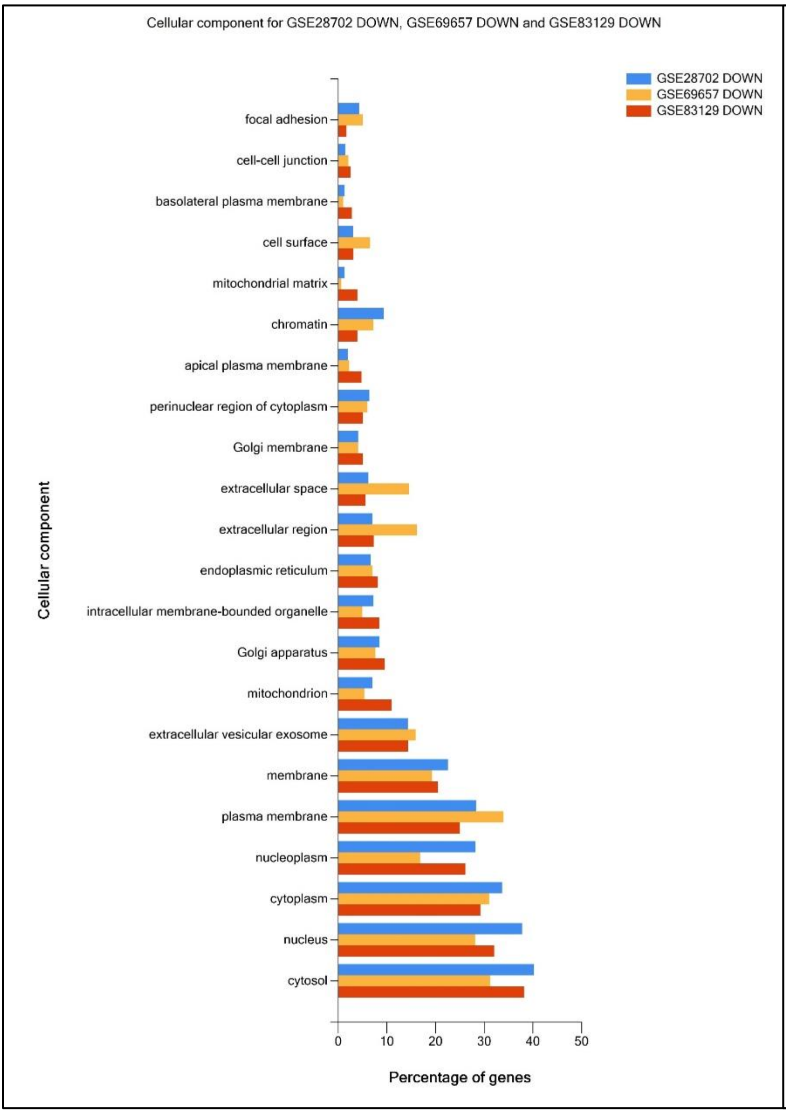

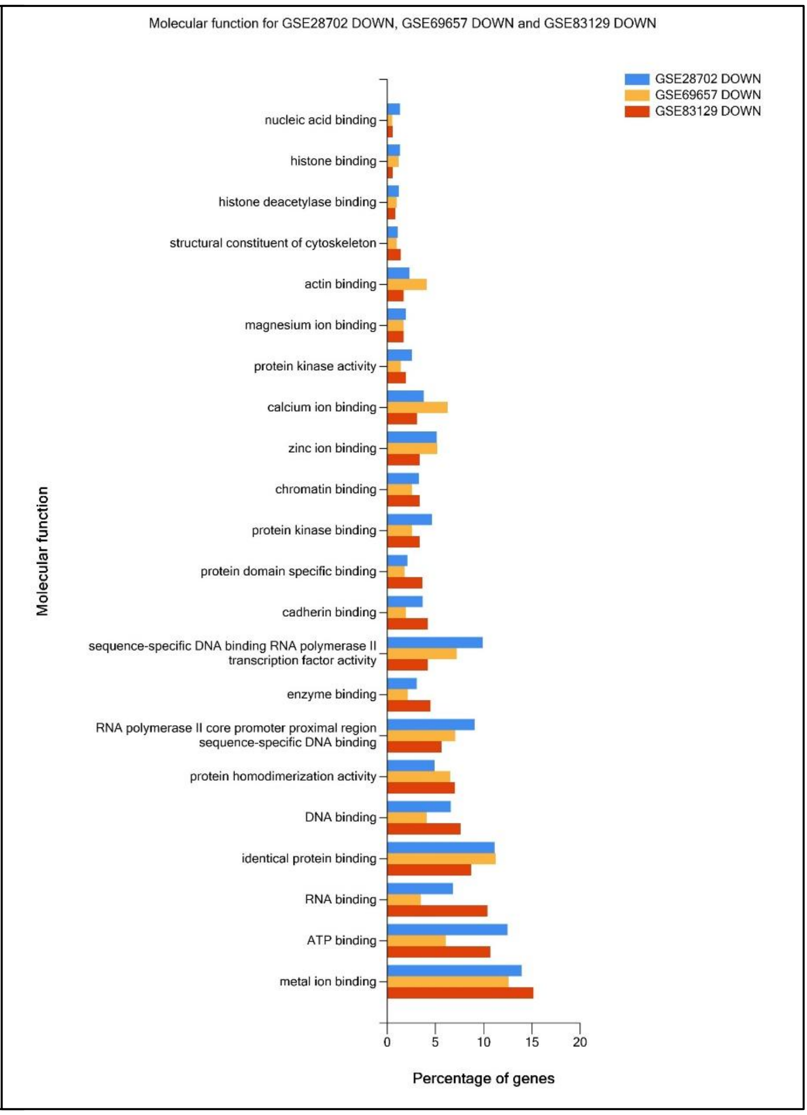

Supplementary S2

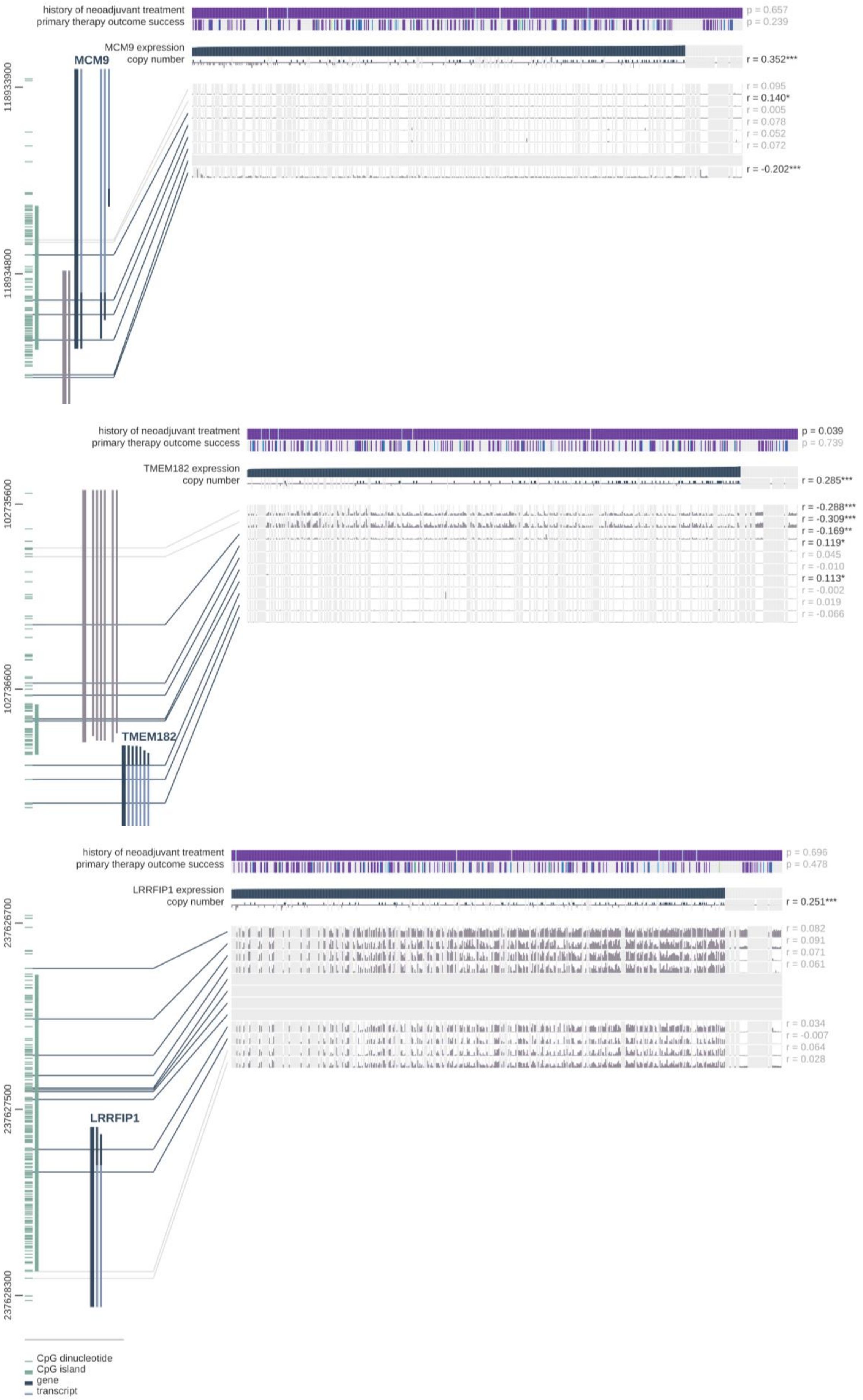

Supplementary S3

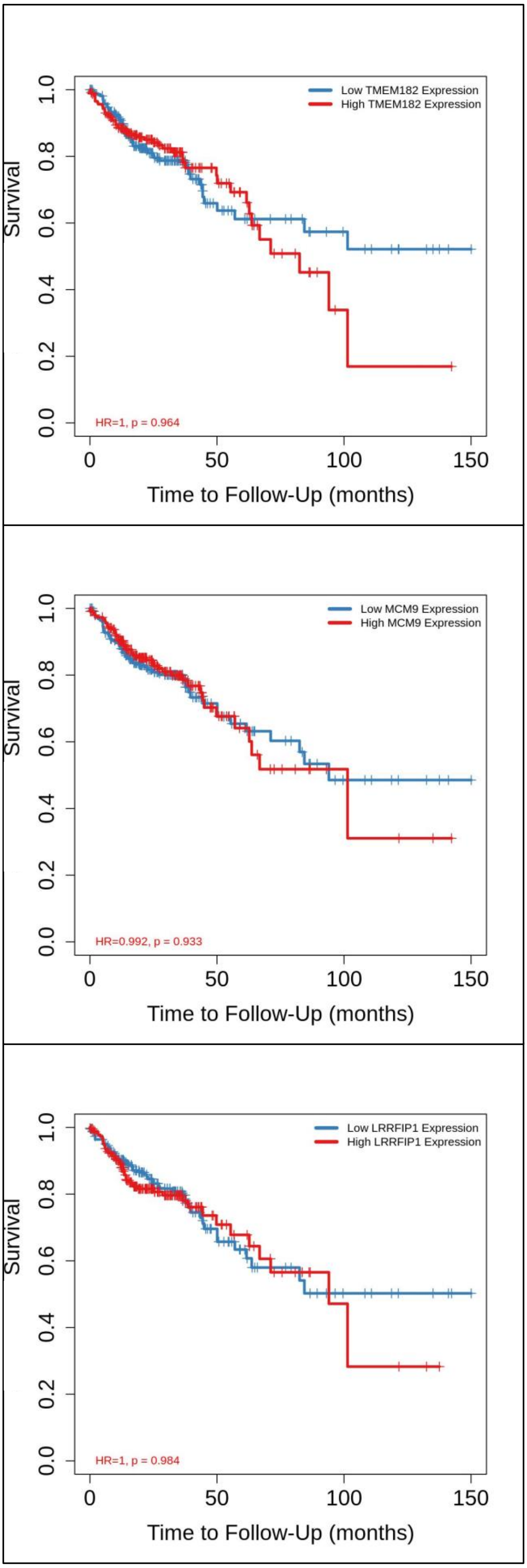

Supplementary S4

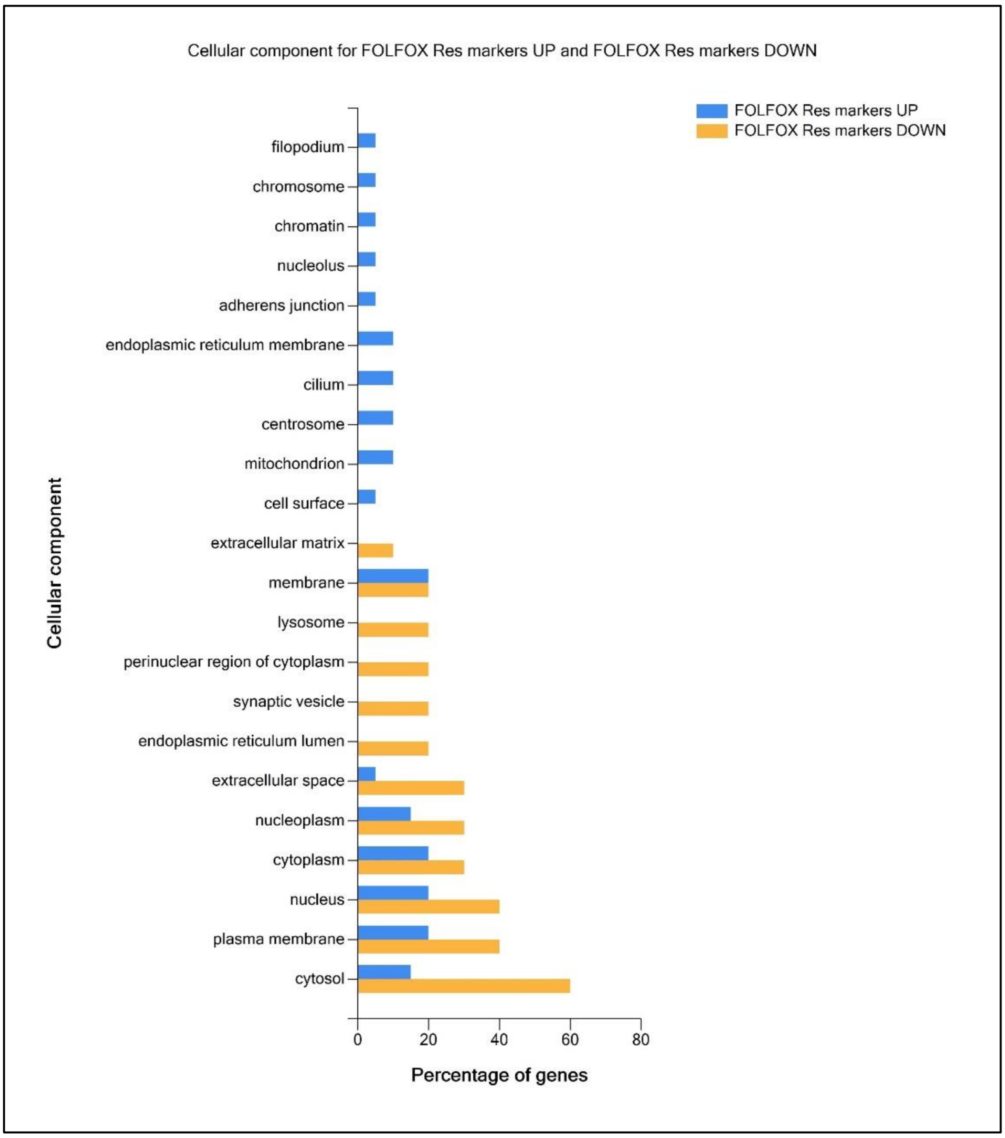

Supplementary S5A

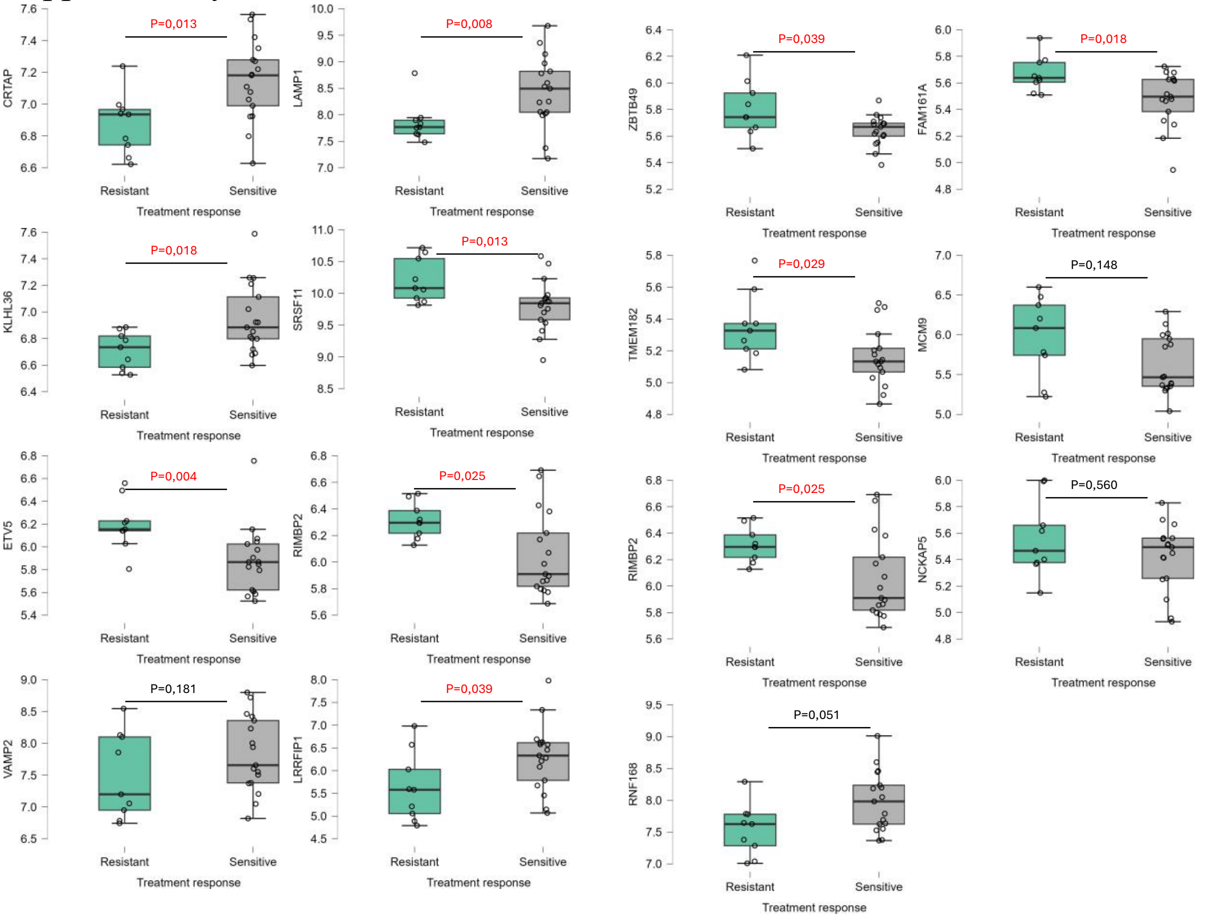

Supplementary S5B

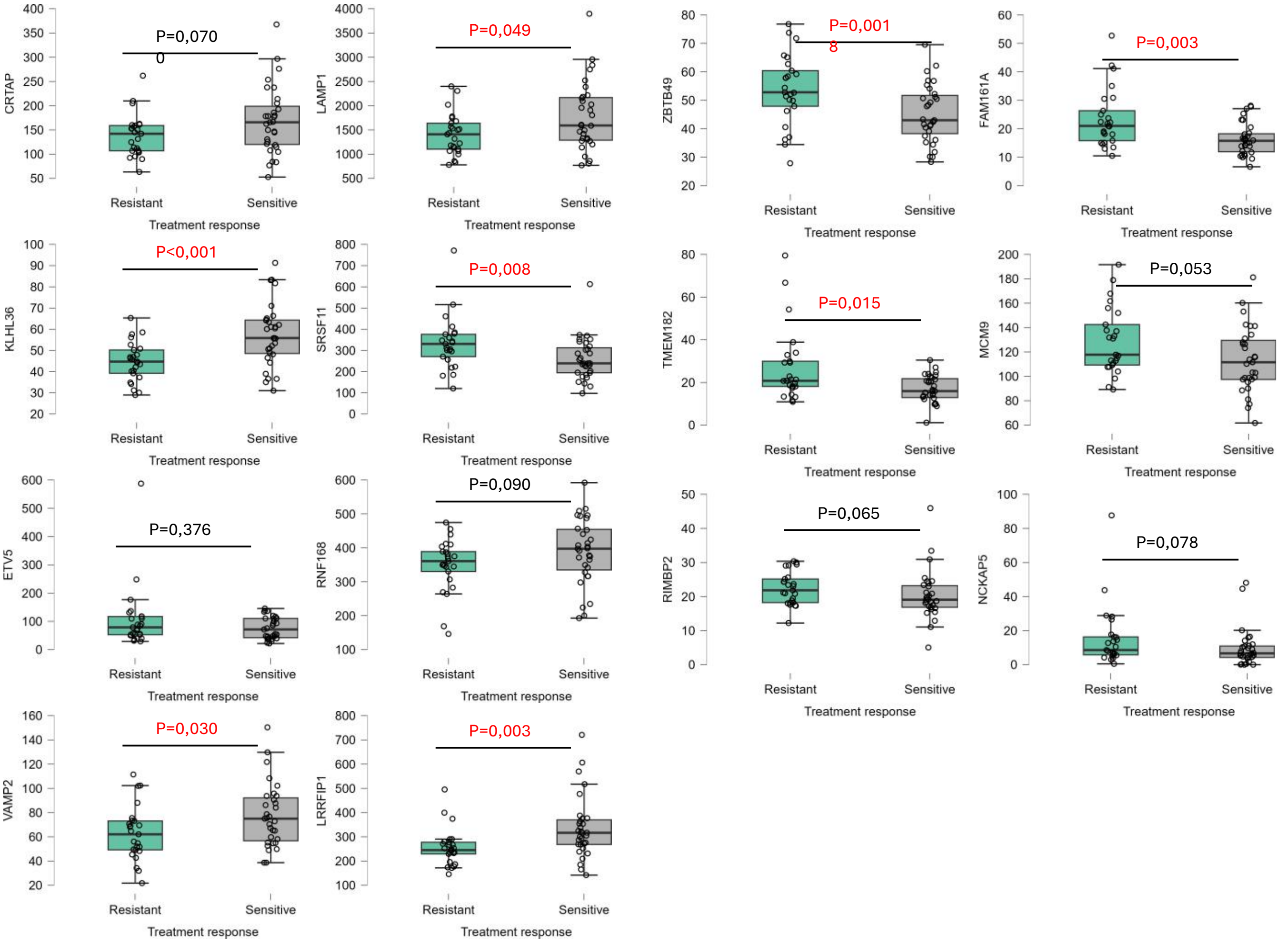

Supplementary S5C

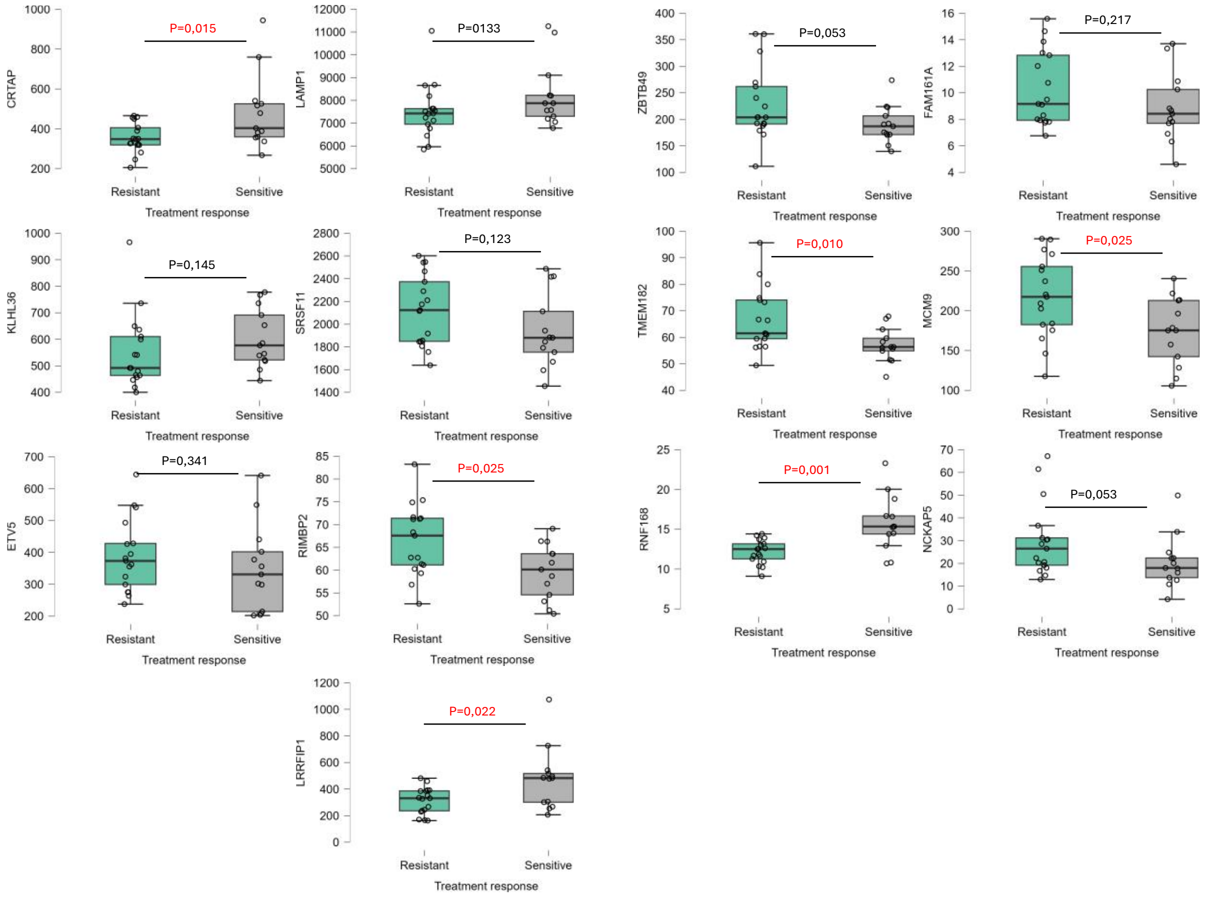

Supplementary S6A

GSE83129

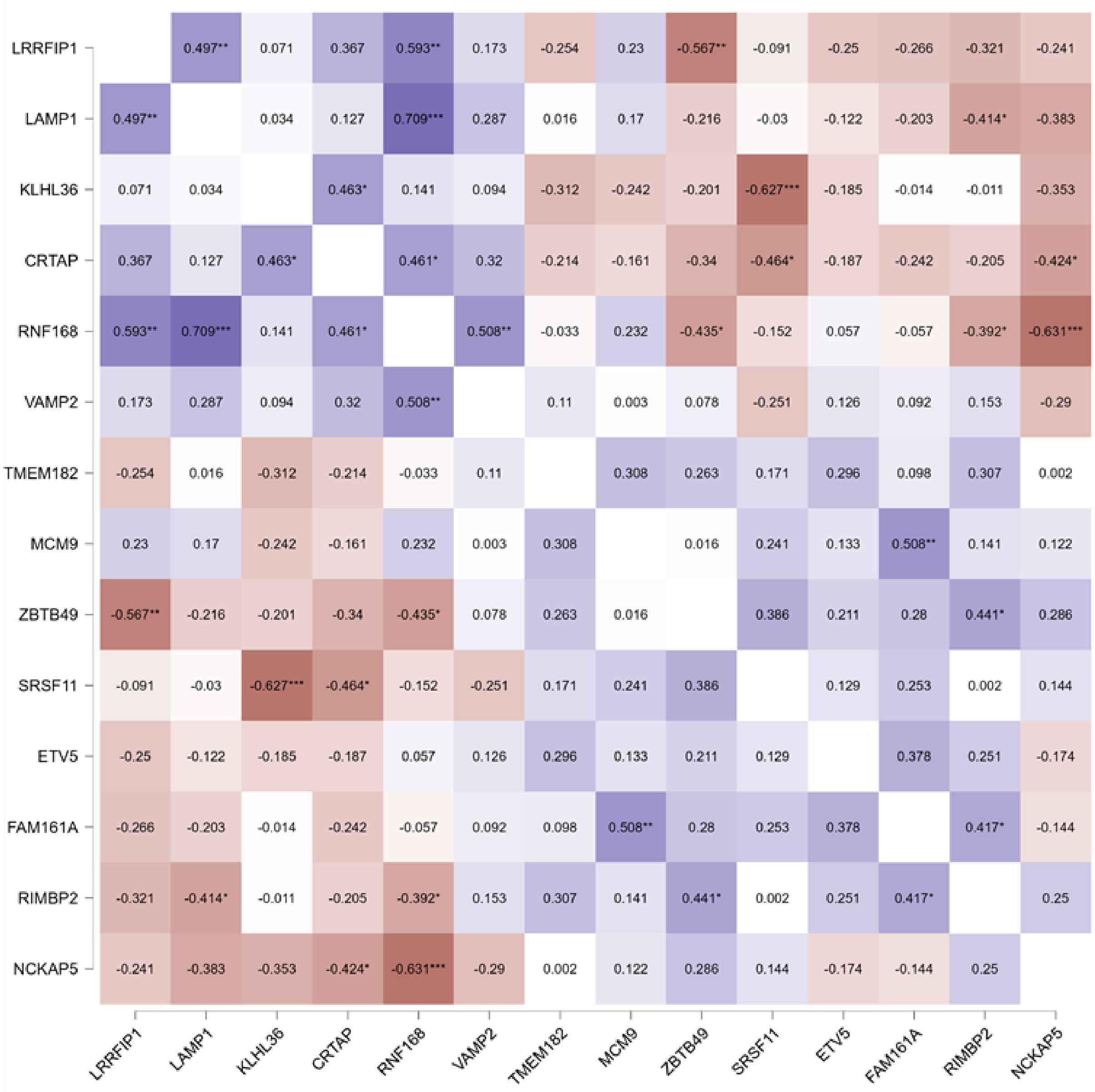

Supplementary S6B.

GSE28702

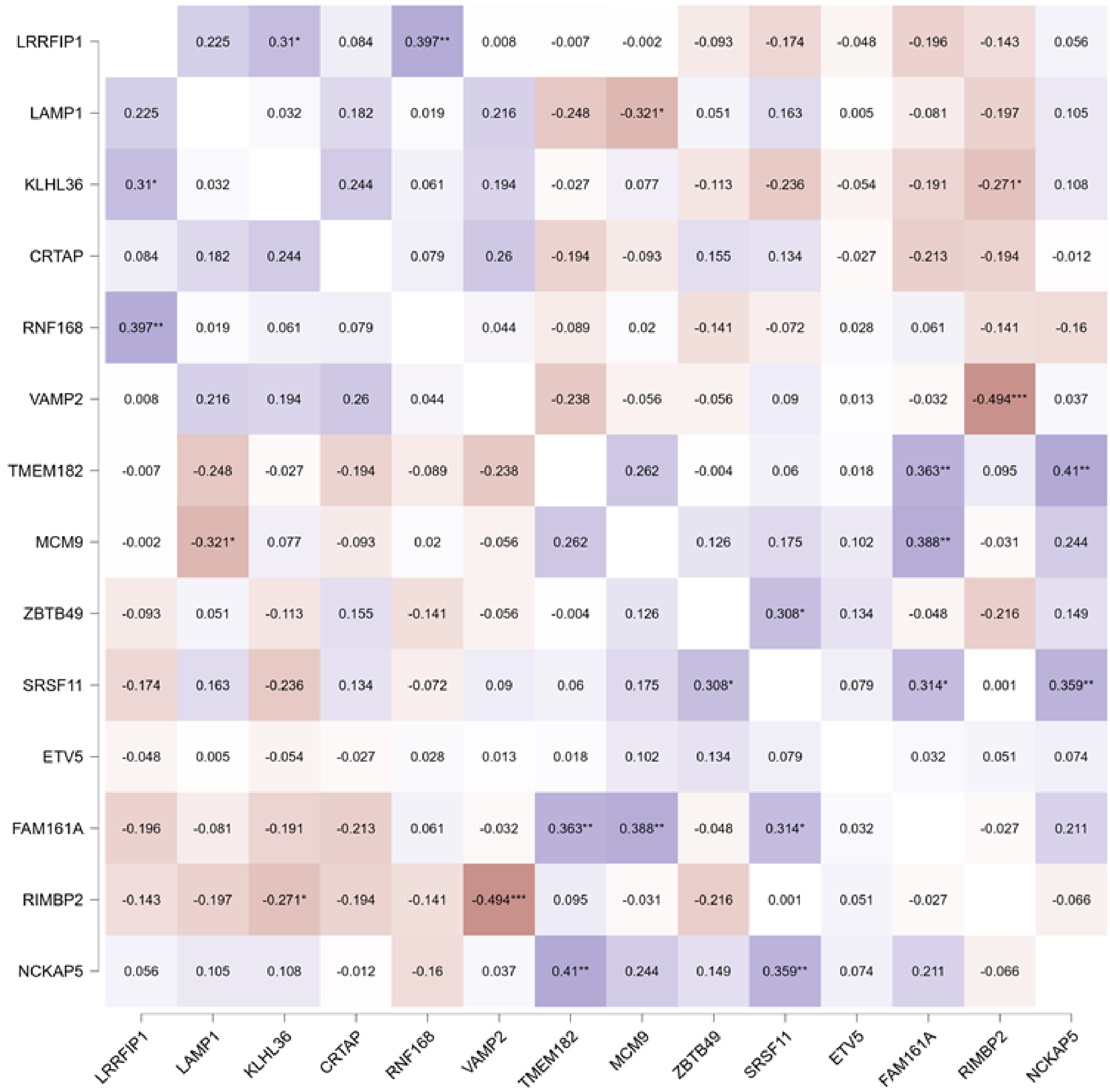

Supplementary S6C

GSE69657

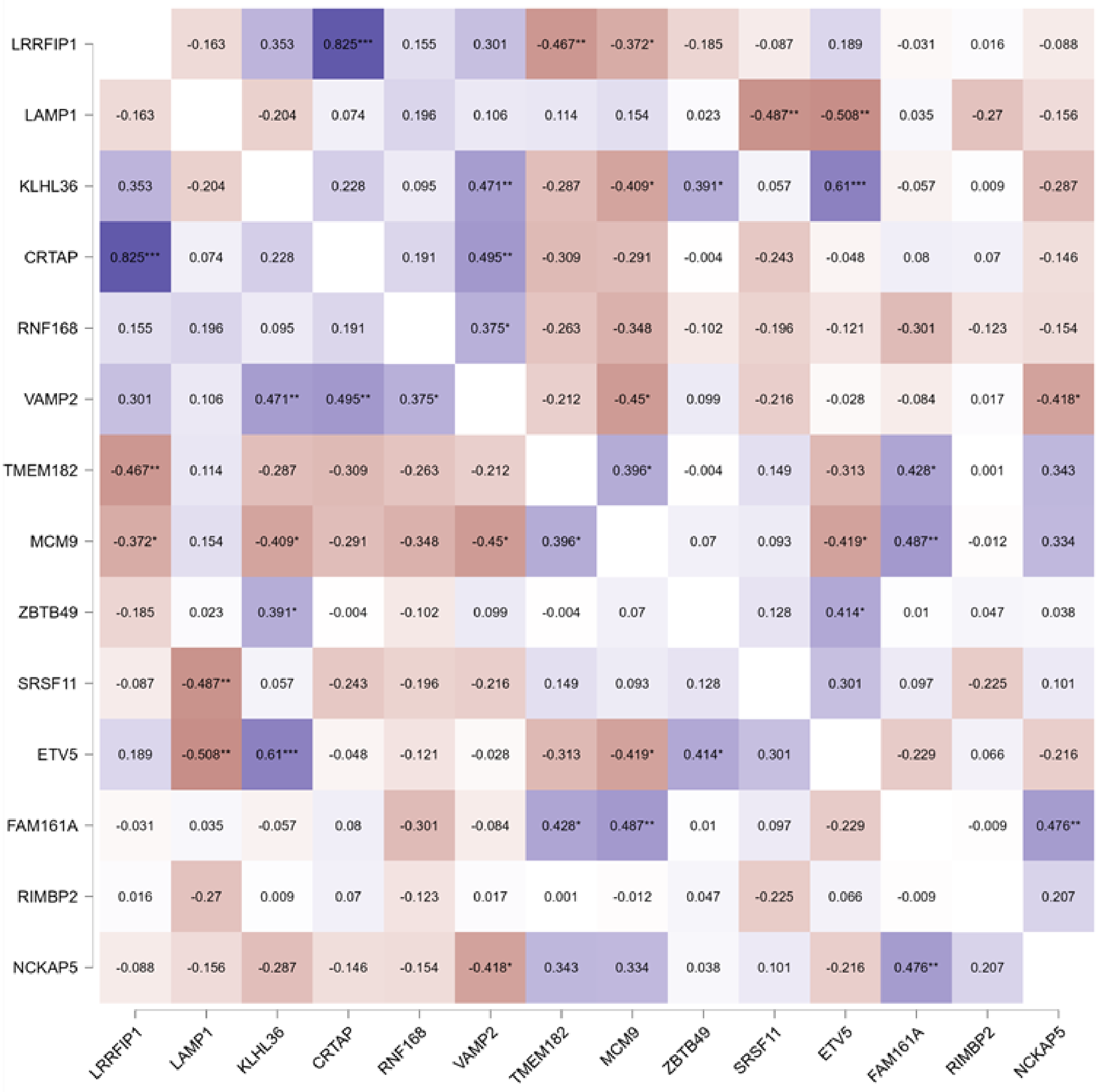

Supplementary S7

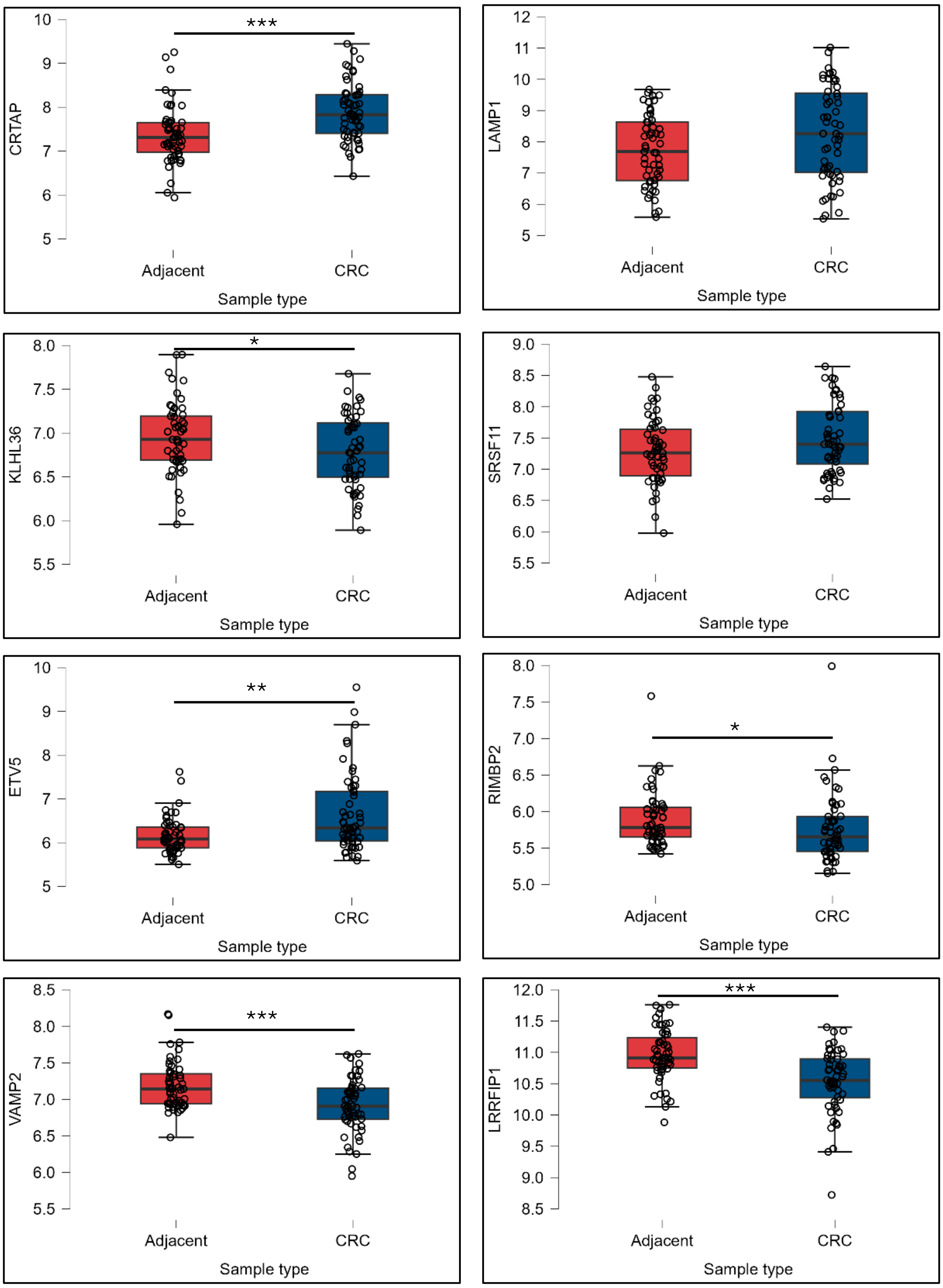

Legends to supplementary figures:

Supplementary S1A Comparative analysis of FOLFOX resistance-related up-regulated A) and down-regulated B) genes functional enrichment. Percentage share of genes enriched in Cellular components (Cc) and Molecular functions (Mf) were calculated and visualized using FunRich software (v3.1.3) supported by Gene Ontology (GO) database.

Supplementary S2. DNA methylation of ***MCM9*****,** ***TMEM182*****, and** ***LRRFIP1*** **promotor sequence.** A single-gene methylation data was obtained from MEXPRESS (https://mexpress.be/) platform and TCGA database on a single-gene level. Correlation significance "r" of corresponding gene expression and methylation of chosen genes is depicted as follows * p>0.05, ** p> 0.005, ***p>0.001.

Supplementary S3. ***MCM9*****,** ***TMEM182*****, and** ***LRRFIP1*** **expression level impact on cumulative survival of CRC patients.** Clinical data obtained from the TCGA database, analysed and visualized using the Human Protein Atlas (www.proteinatlas.org) "pathology" section [15,27] and TIMER2.0 platform (http://timer.cistrome.org) [64]. Presented data used the best expression cut-off suggested by HPA and were composed of respectively *TMEM182*: n=470 "low" expression patients and n=127 "high" expression patients, *MCM9:* n=401 and n=196, *LRRFIP1:* n=410 and n=187. The 5-year survival rate for patients with high expression of *TMEM182* =57% low *TMEM182* expression =65%; high expression MCM9 = 57% and low expression MCM9 = 63%; high expression *LRRFIP1* = 62% and low expression *LRRFIP1*= 61%. The log-rank test p-value for *TMEM182* $p=8.15x10^{-2}$, *MCM9* $p=2x10^{-1}$, and for *LRRFIP1* $p=7,63x10^{-1}$.

Supplementary S4 The percentage share of up- and down-regulated genes functionally enriched in Cellular components (Cc) calculated and visualized using FunRich software (v3.1.3) supported by the Gene Ontology (GO) database.

Supplementary S5ABC Chosen gene expression in Resistant and Sensitive CRC groups from GSE83129 A), GSE28702 B), and GSE69657 C). A normality test (Shapiro–Wilk) was performed, followed by the Mann–Whitney U test with p depicted on each graph. Red – significant, black – not significant.

Supplementary S6ABC. Pearson correlation matrix of FOLFOX resistance-related DEGs. Expression values of mRNA of chosen FOLFOX resistance related up- and down-regulated genes from GSE83129 A), GSE28702 B) and GSE69657 C) were pooled to create Pearson correlation matrix using JASP 0.14.1.0 software; * p>0.05, ** p> 0.005, ***p>0.001

Supplementary S7 Chosen gene expression in CRC samples vs Adjacent tissue from GSE44861. Normality test (Shapiro–Wilk) was performed, followed by the Mann–Whitney U test with p depicted on each graph. * p>0.05, ** p> 0.005, ***p>0.001
